# Multiple Immune Factors Are Involved in Controlling Acute and Chronic Chikungunya Virus Infection

**DOI:** 10.1371/journal.pntd.0003354

**Published:** 2014-12-04

**Authors:** Yee Suan Poo, Penny A. Rudd, Joy Gardner, Jane A. C. Wilson, Thibaut Larcher, Marie-Anne Colle, Thuy T. Le, Helder I. Nakaya, David Warrilow, Richard Allcock, Helle Bielefeldt-Ohmann, Wayne A. Schroder, Alexander A. Khromykh, José A. Lopez, Andreas Suhrbier

**Affiliations:** 1 QIMR Berghofer Medical Research Institute, and the Australian Infectious Diseases Research Centre, Brisbane, Queensland, Australia; 2 School of Medicine/School of Molecular and Microbial Sciences, University of Queensland, Brisbane, Queensland, Australia; 3 Institut National de Recherche Agronomique, Unité Mixte de Recherche 703, Oniris, Nantes, France; 4 School of Pharmaceutical Sciences, University of São Paulo, São Paulo, Brazil; 5 Public Health Virology Laboratory, Department of Health, Queensland Government, Brisbane, Queensland, Australia; 6 Lotterywest State Biomedical Facility Genomics, Royal Perth Hospital, Perth, Western Australia, Australia; 7 School of Veterinary Science, The University of Queensland, Gatton, Queensland, Australia; 8 School of Natural Sciences, Griffith University, Nathan, Australia; Centers for Disease Control and Prevention, United States of America

## Abstract

The recent epidemic of the arthritogenic alphavirus, chikungunya virus (CHIKV) has prompted a quest to understand the correlates of protection against virus and disease in order to inform development of new interventions. Herein we highlight the propensity of CHIKV infections to persist long term, both as persistent, steady-state, viraemias in multiple B cell deficient mouse strains, and as persistent RNA (including negative-strand RNA) in wild-type mice. The knockout mouse studies provided evidence for a role for T cells (but not NK cells) in viraemia suppression, and confirmed the role of T cells in arthritis promotion, with vaccine-induced T cells also shown to be arthritogenic in the absence of antibody responses. However, MHC class II-restricted T cells were not required for production of anti-viral IgG2c responses post CHIKV infection. The anti-viral cytokines, TNF and IFNγ, were persistently elevated in persistently infected B and T cell deficient mice, with adoptive transfer of anti-CHIKV antibodies unable to clear permanently the viraemia from these, or B cell deficient, mice. The NOD background increased viraemia and promoted arthritis, with B, T and NK deficient NOD mice showing high-levels of persistent viraemia and ultimately succumbing to encephalitic disease. In wild-type mice persistent CHIKV RNA and negative strand RNA (detected for up to 100 days post infection) was associated with persistence of cellular infiltrates, CHIKV antigen and stimulation of IFNα/β and T cell responses. These studies highlight that, secondary to antibodies, several factors are involved in virus control, and suggest that chronic arthritic disease is a consequence of persistent, replicating and transcriptionally active CHIKV RNA.

## Author Summary

The largest epidemic ever recorded for chikungunya virus (CHIKV) started in 2004 in Africa, then spread across Asia and recently caused tens of thousands of cases in Papua New Guinea and the Caribbean. This mosquito-borne alphavirus primarily causes an often debilitating, acute and chronic polyarthritis/polyarthalgia. Despite robust anti-viral immune responses CHIKV is able to persist, with such persistence poorly understood and the likely cause of chronic disease. Herein we highlight the propensity of CHIKV to persist long term, both as a persistent viraemia in different B cell deficient mouse strains, but also as persistent viral RNA in wild-type mice. These studies suggest that, aside from antibodies, other immune factors, such as CD4 T cells and TNF, are active in viraemia control. The work also supports the notion that CHIKV disease, with the exception of encephalitis, is largely an immunopathology. Persistent CHIKV RNA in wild-type mice continues to stimulate type I interferon and T cell responses, with this model of chronic disease recapitulating many of the features seen in chronic CHIKV patients.

## Introduction

The arthritogenic alphaviruses comprise a group of globally distributed, mosquito-borne, single-stranded positive-sense RNA viruses that cause sporadic outbreaks of predominantly rheumatic disease. They include the predominantly Afro-Asian chikungunya virus (CHIKV), the primarily Australian Ross River and Barmah Forest viruses, the African o'nyong-nyong virus, the Sindbis group of viruses and the South American Mayaro virus. Symptomatic infection of adults with these alphaviruses is nearly always associated with rheumatic disease, primarily polyarthralgia and/or polyarthritis. The arthopathy can be chronic and debilitating and usually lasts weeks to months, occasionally longer [1]. The largest documented outbreak of CHIKV disease ever recorded began in 2004, resulting in an estimated 1.4–6.5 million cases, mainly in Africa and Asia. Imported cases were reported in nearly 40 countries including Europe, Japan and the USA [1], [2]. The outbreak continues in 2013/2014 with thousands of cases in Papua New Guinea [3] and the Caribbean [4], [5]. At present, no licensed vaccine or particularly effective drug is available for human use for any alphavirus, although analgesics and non-steroidal anti-inflammatory drugs can provide relief from symptoms [1], [6].

Alphavirus infections *in vivo* result in a brief, usually 5–7 day viraemia, which is primarily controlled by IFNα/β initially, and subsequently by anti-viral antibodies. Infection of genetically modified mice defective in IFNα/β responses have illustrated that a rapid early induction of IFNα/β is required to control the acute viraemia and protect against mortality [7], [8], [9], [10]. Antibodies are also well recognized as mediating protection, with anti-viral antibodies [11], [12], [13], [14] and antibody-based vaccines [15], [16], [17], [18] being developed as potential prophylactic interventions. An important role for CD4 T cells in driving CHIKV arthritis was recently established [19], [20]. However, the role of T cells in controlling alphaviral viraemia remains controversial with recent reports suggesting they have no role [20], [21], whilst early literature described a role for T cells in cross protection between different alphaviruses [22], [23], [24]. NK cells appear to have a protective role for alphaviral infections in some settings [25], but not others [26], with NK cells also implicated in arthritic disease [27], [28].

Alphaviruses have a well recognized propensity to establish persistent infections *in vitro* [29], [30], [31], [32], [33] and *in vivo* [34], [35], [36], [37], with such persistence in joint tissues likely responsible for chronic arthritic disease [38], [39], [40]. How such post-viraemia persistence is achieved in the face of robust anti-viral antibody and T cell responses remains a matter of considerable speculation [32], [41], [42], [43], [44], [45], [46]. Antibodies and T cell IFNγ are believed to be involved in the ultimate clearance of persistent Sindbis virus from neurons [47]. However, knowledge regarding the nature of persistent arthritogenic alphavirus infections, and the inflammatory responses stimulated by them, currently remains limited [38], [40].

We recently developed an adult C57BL/6 (wild-type) mouse model of CHIKV infection and arthritis that mimics many aspects of human disease [48]. Herein we use this infection model in a series of genetically modified mouse strains deficient in one or more immune responses to explore the contribution of B, T and NK cells and the non-obese diabetic (NOD) background to (i) protection against CHIKV viraemia and (ii) promotion of arthritic disease. We also show, consistent with human and monkey data [38], [40], that in C57BL/6 mice, CHIKV RNA and protein persists for extended periods and continues to stimulate innate and adaptive immune responses.

## Materials and Methods

### Mice

The mice strains used in this study were: (i) NRG (B, T and NK cell deficient on a NOD background), NOD.Cg-*Rag1^tm1Mom^ Il2rg^tm1Wjl^*/SzJ, NOD-congenic mice harboring the *Rag1^null^* mutation and the *IL2rγ^null^* mutation (JAX); (ii) NOD, NOD/ShiLtJ (non-obese diabetic mouse) (JAX); (iii) Rag2/Il2rg (B, T and NK cell deficient on a B6 background), B10; B6-*Rag2^tm1Fwa^ Il2rg^tm1Wjl^* (Taconic, Hudson, NY), (iv) Rag1^−/−^ (B and T cell deficient on a C57BL/6 background), B6.129S7-*Rag1^tm1Mom^*/J (JAX); (v) µMT (B cell deficient on a C57BL/6 background, no expression of membrane-bound IgM), B6.129S2-*Igh-6^tm1Cgn^*/J (JAX); (vi) MHCII^Δ/Δ^ (CD4 T cell deficient, no class II MHC on a C57BL/6 background) [49]; (vii) FcγR^−/−^ mice (Fc gamma receptor deficient on a C57BL/6 background), B6.129P2-*Fcer1gtm1Rav* N12 (Taconic). All strains (except FcγR^−/−^) were bred at the QIMR Berghofer animal house facility. C57BL/6 mice were purchased from Animal Resources Center (Canning Vale, WA, Australia). All animals were handled in accordance with good animal practice as defined by the National Health and Medical Research Council of Australia. All experiments were approved by the QIMR Berghofer animal ethics committee (P1060 A0705-603M).

### Virus infections, viraemia determination and measurement of foot swelling

The Reunion Island isolate (LR2006-OPY1) of CHIKV is a primary isolate obtained from the recent outbreak in Reunion Island and was grown in C6/36 cells, inoculated into mice, and serum viraemia determined as described previously using a modified CPE-based assay on Vero cells [8], [48]. Female mice were used with an age range of 6–12 weeks (mean age of each group was 8–10.5 weeks); we have not observed significant differences in foot swelling for mice within this age range using this model (Table S1 in Text S1). Mice were inoculated with 10^4^ CCID_50_ of virus subcutaneously (s.c.) into the dorsal side of both hind feet, toward the ankle. Blood was collected from the tail vein into MiniCollect tubes (Greiner Bio-One GmbH, Kremsmunster, Austria) and viral titers expressed as log_10_ 50% cell culture infectivity dose (CCID_50_) (method of Spearman and Kaber). Foot swelling was measured using digital Vernier calipers and is presented as a group average of the percentage increase in foot height times width for each foot compared with the same foot on day 0 (i.e. n = 12 feet means n = 6 mice unless stated otherwise).

### Cytokine/chemokine analyses

Serum cytokine protein levels were analyzed using the BD Cytometric Bead Array Bioanalyzer system (Becton Dickinson, Franklin Lakes, NJ) and IFNα levels were determined by Mouse IFN-alpha FlowCytomix Simplex (eBioscience, San Diego, CA, USA) according to the manufacturer's instructions.

### Vaccination and proliferation assays

Mice were vaccinated s.c. with 10 µg of inactivated CHIKV as described [48]. Standard proliferation assays using tritiated thymidine uptake were undertaken using splenocytes isolated 3 weeks post vaccination. Briefly, splenocytes (2.5×10^5^ cells/96 well, 6 replicates) were cultured with 10 µg/ml of inactivated CHIKV [48] for 3 days, tritiated thymidine was then added and cells harvested the next day onto a MicroBeta Filtermat-96 A using the FilterMate™ Cell Harvester (PerkinElmer). Radioactivity was measured using the MicroBeta Liquid Scintillation Counter (PerkinElmer).

### ELISA assays

Anti-CHIKV IgG2c and IgG1 antibody titers were determined by standard isotype-specific ELISA using ELISA plates coated with inactivated CHIKV as described [15].

### Anti-CHIKV anti-serum

Anti-CHIKV anti-serum was generated by infecting C57BL/6 mice with CHIKV and after 10 weeks vaccinating them with 10 µg of inactivated CHIKV [48]. Serum was harvested after 2 weeks and had an end point neutralization titer of 1/2560 determined as described [15].

### Histology

Tissues were fixed in 10% neutral buffered formalin, feet were decalcified (15% EDTA in 0.1% phosphate buffer over 10 days), tissue was embedded in paraffin wax, and 6 µm-thick sections were cut and stained with hematoxylin-eosin. Sections were digitally scanned using Scan Scope XT digital slide scanner (Aperio, Vista, CA). Image analyses were undertaken using Aperio ImageScope Software (v10) and the Positive Pixel Count v9 algorithm (default settings).

### Real time quantitative RT-PCR

Quantitative real time RT-PCR was undertaken as described [48]. Briefly, feet and spleen were stored in RNAlater solution (Ambion, Austin, TX, USA), placed in TRIzol (Life Technologies, Carlsbad, CA, USA) and homogenization using steel balls and TissueLyser (Qiagen) at 25 Hertz for 6 min. on ice. cDNA was then generated using Superscript III (Invitrogen) and random hexamer oligonucleotides. Real-time PCR analysis used the following primers (5′ to 3′): CHIKV E1 F AGCTCCGCGTCCTTTACC, R CAAATTGTCCTGGTCTTCCTG; ISG54 F CTCTCTGGAGCAAGCCATTC, R GCCATTGCTTGGTTTTTATG. Quantitative real-time PCR (qRT-PCR) was performed in a reaction consisting of 1 µl of cDNA, 10 µl of SYBR green Super mix-UDG (Invitrogen), 1 µl BSA, 6 µl H_2_O, and 1 µl of 10 µM of forward and reverse primers. cDNA was amplified and PCR products were detected using Rotorgene 6000 (Corbett Research, Mortlake, Australia) under the following cycling conditions: one cycle of 50°C for 2 min, one cycle of 95°C for 2 min, 45 cycles of 94°C for 5 sec, 60°C for 10 sec and 72°C for 30 sec. Data were analyzed using Rotor-Gene Real Time Analysis software (Corbett Research, Australia). Each sample was analyzed in duplicate and normalized to RPL13A mRNA as described [48].

Negative-strand specific qRT PCR was undertaken essentially as described [50]. cDNA was synthesized as described [48] with the exception that random hexamer oligonucleotides were substituted with 10 pg of a primer, that comprised a tag sequence linked to a CHIKV nsP1 sequence (5′-*GGCAGTATCGTGAATTCGATGC*GACACGGAGACGCCAACATT-3′; tag sequence in italics). qRT PCR used a forward primer with the tag sequence (5′-AATAAATCATAA *GGCAGTATCGTGAATTCGATGC*-3′) and a reverse primer from nsP1 (5′-AATAAATCATAAGTCTGCTCTCTGTCTACATGA-3′), with flap sequences (underlined) added to increase fluorescent signal strength [50].

### Statistics

Analyses were performed using IBM SPSS Statistics (version 19). The t test was used if the difference in the variances was <4, skewness was >−2, and kurtosis was <2. Where the data was non-parametric and difference in variances was <4, the Mann Whitney U test was used, if >4 the Kolmogorov-Smirnov test was used.

### Microarray studies

Microarray studies were performed essentially as described [19]. RNA from feet taken day 0 was compared with RNA from feet taken day 30 post infection, two microarrays were undertaken for each time point. Probe sets that did not represent known genes were removed and only expressed genes with a mean log_2_ expression≥6 and variance >0.1 across all 4 samples were included. A t-test was performed to compare gene expression between day 0 and day 30 samples for the 4,805 remaining genes. Genes where p<0.05 were considered differentially expressed. Differentially expressed genes were analyzed using web-based Ingenuity pathway analysis (IPA) using canonical pathway analysis [19] and the upstream regulator function [51].

## Results

### CHIKV viraemia in B cell, T cell and/or NK cell deficient mice

The following mouse strains were infected with CHIKV and their viraemias were monitored over time; (i) C57BL/6 mice, (ii) non obese diabetic (NOD) mice, (iii) MHCII^Δ/Δ^ mice (MHCII deficient mice on a C57BL/6 background), which lack of functional Th cells and thus no T cell help for B cells [49], (iv) µMT mice (B cell deficient on a C57BL/6 background), (v) Rag1^−/−^ mice (B and T deficient on a C57BL/6 background), (vi) Rag2/Il2rg mice (B, T and NK cell deficient on a C57BL/6 background), (vii) NRG mice (B, T and NK cell deficient on a NOD background). The mice and their characteristics are fully described in Table S2 in Text S1.

**Figure 1:**
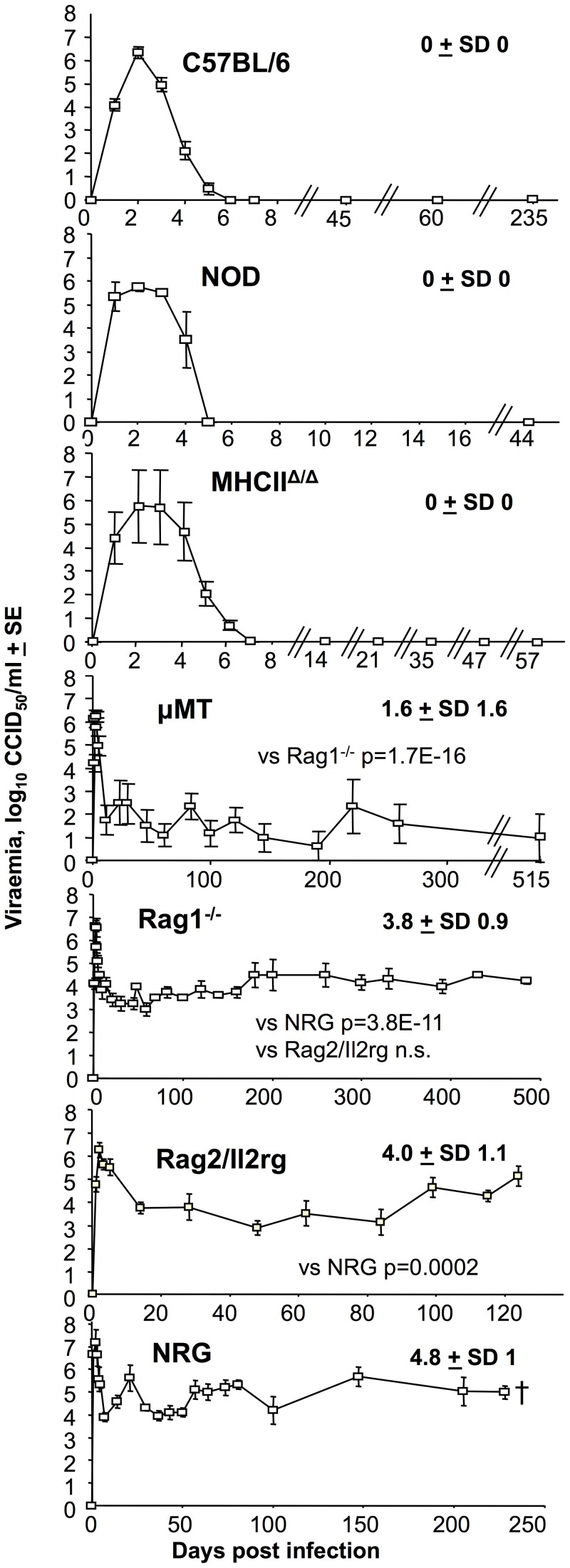
Viraemias in different mouse strains; B cell deficiency results in persistent “set point” viraemias. The viraemias for the indicated mouse strains at the indicated times are shown (strains ranked best to worst in terms of ability to control the viraemia); C57BL/6 (n = 43–50 mice before day 6, 3–9 thereafter; data from 8 independent experiments; not all time points were tested in each experiment), NOD (n = 6, except day 44 where n = 4, data from 2 independent experiments), MHCII^Δ/Δ^ (CD4 T cell deficient) (n = 9–16 day 0–6, n = 4–7 thereafter; data from 4 independent experiments), µMT (B cell deficient) (n = 6–10; data from 2 independent experiments), Rag1^−/−^ (B and T cell deficient) (n = 7–16; data from 3 independent experiments), Rag2/Il2rg (B, T and NK cell deficient) (n = 4), NRG (B, T and NK cell deficient on a NOD background) (n = 5–13 day 0–37, n = 3–9 thereafter; data from 3 independent experiments). Set-point viraemia levels for each mouse strain are indicated (bold, top right) and represent the mean (log_10_CCID_50_/ml of serum +SD) of all viraemia measurements taken ≥10 days post infection. For B cell deficient mice (bottom 4 panels), statistical comparisons (by t test) of set-point viraemia levels (*e.g.* p = 3.8E–11 for comparison of Rag1^−/−^ with NRG) used all viraemia measurements taken ≥10 days post infection. All mice were on a C57BL/6 background except the NOD and NRG mice.

C57BL/6, NOD and MHCII^Δ/Δ^ mice were able efficiently to control viraemia by day 5–7 (Fig. 1, C57BL/6, NOD, MHCII^Δ/Δ^); these mouse strains all have B cells. The results for C57BL/6 and MHCII^Δ/Δ^ mice are consistent with previous reports [19], [20], [48]. The mean viraemia in MHCII^Δ/Δ^ mice was ≈2.5 logs higher than in C57BL/6 mice on day 4 (p = 0.024, Kolmogorov Smirnov test), ≈1.5 logs higher on day 5 (not significant), and ≈0.5 logs higher on day 6 (not significant), suggesting a slight delay in viraemia control in these mice (see also below for antibody responses in these mice).

In mouse strains lacking B cells, CHIKV viraemias peaked on days 2–3, and then settled to relatively constant levels that were distinct in several mouse strains (Fig. 1, bottom 4 graphs; µMT^−/^, Rag1^−/−^, Rag2/Il2rg and NRG). Persistent CHIKV infection in Rag1^−/−^ and µMT^−/−^ mice has been reported previously [36], [37]. The leveling out of viraemias in these mice (Fig. 1) is reminiscent of peripheral blood set-point viral loads described for HIV, with the set-point levels deemed to be a reflection of functional anti-viral immunity [52]. Applying this concept to the data presented herein (Fig. 1), the CHIKV set-point viraemias were determined by calculating the mean of all viraemia measurements taken on and after day 10 post infection (Fig. 1, values in bold +SD). The set-point viraemia levels were (lowest to highest) µMT<Rag1^−/−^ =  Rag2/Il2rg <NRG, with each “<” representing statistically significant differences (Fig. 1, p values). These results suggest that T cells contribute to suppression of viraemia as the set-point viraemia was ≈2 logs higher in Rag1^−/−^ mice (B and T cell deficient) than in µMT mice (B cell deficient) (Fig. 1, Rag1^−/−^ vs. µMT). NK cells do not appear to play a major role in viraemia control as the set-point viraemia in Rag1^−/−^ mice (B and T cell deficient) and Rag2/Il2rg mice (B, T and NK cell deficient) was not significantly different (Fig. 1, Rag1^−/−^ vs. Rag2/Il2rg). The NOD background (in addition to B, T and NK cell deficiency) further increased the set-point viraemia, with NRG mice showing a significant mean ≈0.8 log higher level than Rag2/Il2rg mice (Fig. 1, NRG vs. Rag2/Il2rg). The NOD background has defects in a number of innate immune activities that might be responsible for this difference [53], [54], [55].

### MHCII^Δ/Δ^ mice generate MHC II-independent anti-viral IgG2c responses

**Figure 2:**
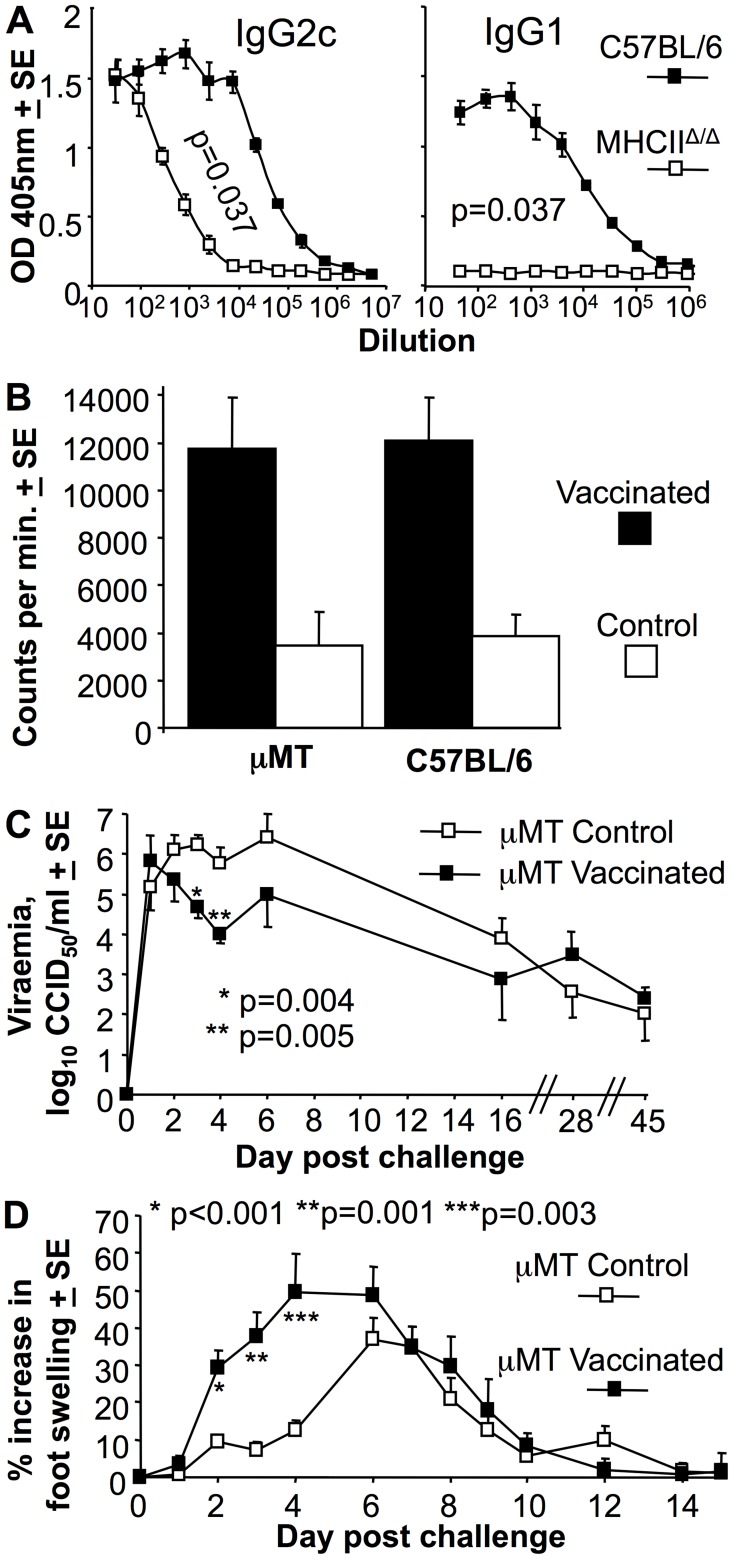
MHCII^Δ/Δ^ mice generate IgG2c responses and vaccinated µMT mice show lower early viraemia, but exacerbated arthritic disease. (A) MHCII^Δ/Δ^ and C57BL/6 mice were analyzed for CHIKV-specific IgG2c and IgG1 levels by ELISA at 21 days post-infection (n = 4 per group). Statistics by Kolmogorov-Smirnov test comparing 50% end point titers. (B) µMT and C57BL/6 mice were inoculated with 10 µg of inactivated CHIKV vaccine (Vaccinated) or PBS s.c. (Control). Splenocytes were harvested day 21 post infection and used in a standard proliferation (tritiated thymidine incorporation) assay using inactivated CHIKV as antigen. Shown is the mean of 2 independent experiments both of which showed significant differences between vaccinated and Control for each mouse strain (µMT p = 0.013 and 0.043; C57BL/6 p = 0.037 and 0.027, statistics by Kolmogorov-Smirnov and t test (n = 4–5 per group). (C) Viraemia following standard CHIKV challenge of µMT mice that had received PBS (µMT Control) or 10 µg of inactivated CHIKV vaccine s.c. (µMT vaccinated) 3 weeks previously. Statistics by Mann Whitney U or Kolmogorov-Smirnov tests, (n = 6–10 mice per group; data from 2 independent experiments). (D) Foot swelling following standard CHIKV challenge of µMT mice that had received PBS or CHIKV vaccine as for C. Statistics by Kolmogorov-Smirnov tests (n = 8–20 feet per group; data from 2 independent experiments).

MHCII^Δ/Δ^ mice are defective for T cell help in B cell IgG class switching and have a dearth of CD4^+^ Th cells [49]. Analysis of the antibody responses in these mice showed that following CHIKV infection, MHCII^Δ/Δ^ mice generated no anti-viral IgG1 responses, but did make anti-viral IgG2c responses, albeit at about ≈100 fold lower titers than C57BL/6 mice (Fig. 2A). CD4^−/−^ mice also show reduced anti-CHIKV IgG1 and IgG2c responses following CHIKV infection [37]; however, CD4-negative MHC II-restricted T cells in these mice retain immunoglobulin isotype class switching activity [56]. MHCII-restricted CD4 T cell-independent IgG2c production has been shown previously to be reliant on IFNα/β signaling in B cells [57], with abundant IFNα/β production well described for CHIKV infections [8]. MHCII-restricted CD4 Th cells thus appear to be required for IgG1 and high titer IgG2c anti-viral responses after CHIKV infection.

MHCII^Δ/Δ^ mice were able effectively to control the viraemia by day 5 (Fig. 1, MHCII^Δ/Δ^). Whether this was due to IgM responses (intact in MHCII^Δ/Δ^ mice) or IgG2c responses remains unresolved, with IgG responses detected in mice using sensitive techniques as early as day 3 post viral infection [58].

### Vaccination of µMT mice suppressed acute viraemia but exacerbated arthritis

The significant ≈2 log difference in set-point viraemia between Rag1^−/−^ and µMT mice (Fig. 1) suggested that T cells play a role in suppressing viraemia (with this being clearly discernable when B cells are absent). CD8 T cells have been shown not to influence viraemia in a Ross River virus mouse model [32] and not to influence viraemia and disease in a CHIKV mouse model [20], [32], suggesting CD4 T cells are likely involved. To further investigate the role of T cells in viraemia control (in the absence of the dominant role of antibodies), B cell deficient µMT mice were vaccinated with an inactivated (non-adjuvanted) CHIKV whole-virus vaccine. This vaccine was previously shown to provide complete protection against CHIKV viraemia and foot swelling (arthritis) in C57BL/6 mice [48]. Vaccinated µMT mice generated similar levels of CHIKV-specific T cell responses to C57BL/6 mice, as measured by standard proliferation assays using inactivated virus as antigen (Fig. 2B). A parallel group of vaccinated and control (PBS-vaccinated) µMT mice were challenged with CHIKV. Vaccinated mice showed a significant ≈1 and ≈1.5 log lower viraemia on days 3 and 4, respectively, compared with control µMT mice. This effect was lost at later time points (Fig. 2C), by which time the control µMT mice would presumably have generated CHIKV-specific T cells in response to the infection. Given unadjuvanted, killed, whole-virus vaccines are generally poor at inducing CD8 T cells [59] and CD4 T cell recall responses usually peak around day 4 [60], this experiment provides further support for an antibody-independent role of CD4 T cells in CHIKV viraemia suppression.

Following challenge, the vaccinated µMT mice showed much earlier and higher foot swelling than unvaccinated µMT mice (Fig. 2D, Control). This observation is consistent with the notion that CD4 T cells have an important immunopathological role in arthritis [19], and highlights a potential risk if a vaccine were to induce T cell responses, but inadequate antibody responses.

### Adoptively transferred antibodies clear virus for only a brief period in Rag1^−/−^ and µMT mice

**Figure 3:**
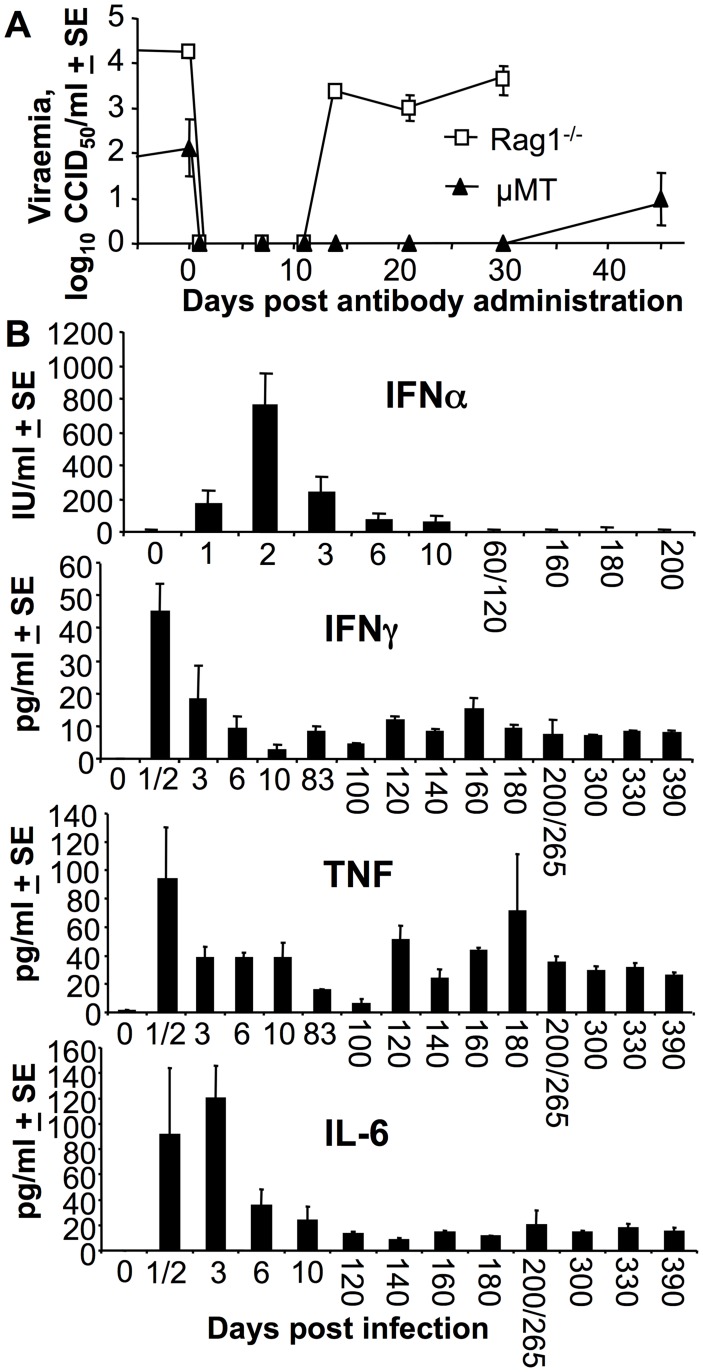
Antibodies were unable to clear virus from persistently infected Rag1^−/−^ and µMT mice, and IFNγ, TNF and IL-6 levels were persistently elevated in persistently infected Rag1^−/−^ mice. (A) Rag1^−/−^ mice day 485 post infection (n = 4) and µMT mice day 550 post infection (n = 4) were given 200 µl immune serum (neutralization titer 1/2560) i.p. on day 0 and viraemia determined on the indicated days. (B) Serum cytokine levels measured in Rag1^−/−^ mice at the indicated times post-infection (n = 3/4 mice per time point). (Data for IFNα day 1 & 2 and day 60 & 120, and day 200 & 265 for the other cytokines were combined, as n = 2 for each one of these times).

Adoptive transfer of anti-viral antibodies has been suggested as both prophylactic and therapeutic interventions for CHIKV [11], [12], [13], [14]. To gain insights into how effective such treatments might be, Rag1^−/−^ and µMT mice persistently infected for >480 days, were treated with mouse polyclonal anti-CHIKV anti-serum. The viraemia became undetectable for 10 and 30 days in Rag1^−/−^ and µMT mice, respectively, but then reappeared thereafter to levels seen prior to antibody administration (Fig. 3A). Passive transfer of antibody was thus unable to clear the virus permanently from these mice, an observation that is consistent with the inability of robust anti-viral humoral immunity to clear persistent virus and/or viral RNA from infected monkeys [38] and humans [40]. In these B cell deficient mice, the adoptive transfer of antibodies worked for only a limited period, consistent with the limited serum half-life of adoptively transferred antibodies [1].

### Persistent viraemia in Rag1^−/−^ mice was associated with elevated serum TNF and IFNγ

To determine what cytokines might be implicated in limiting the viraemia in B and T cell deficient mice, serum cytokine levels were measured in persistently infected Rag1^−/−^ mice. Although acute induction of serum IFNα was observed, levels did not remain elevated despite the ongoing viraemia (Fig. 3B, IFNα). The well described tight control (and thus transient) production of IFNα/β [61], [62] thus appeared to be largely retained in persistently viraemic Rag1^−/−^ mice. In contrast to IFNα/β, serum IFNγ, TNF and IL-6 were persistently up-regulated in persistently infected Rag1^−/−^ mice (Fig. 3B, IFNγ, TNF, IL-6), with IFNγ and TNF previously shown to have anti-alphaviral activities [63], [64]. Elevated levels of CCL2/MCP-1 (a chemokine with no antiviral activity against CHIKV [51]) were also seen, peaking at ≈1000 pg/ml day 1 and settling to a constant level of 200+13.4 pg/ml after day 3. No IL-12 was detected.

### Persistent virus recovered from Rag1^−/−^ mice

The ability of alphaviruses to acquire mutations and better evade the antiviral effects of IFNα/β have been reported [29], [65], with CHIKV and other alphaviruses having evolved strategies to counter the host's type I and II interferon responses [66], [67]. Virus isolated from Rag1^−/−^ mice on 100 behaved no differently from parental virus (with respect to viraemia and foot swelling) when isolated from blood, expanded in C6/36 cells, and used to infect C57BL/6 mice (S1 Figure A in S1 Text). Virus isolated from three Rag1^−/−^ mice day 429 post infection also did not show consistent or significant viraemia differences from parental virus in C57BL/6 mice (S1 Figure A in S1 Text). CHIKV thus appears unable to evade further (via adaptive mutations) the innate factors that maintain the viraemia at the set-point level in Rag1^−/−^ mice. One might speculate that TNF [64] (rather than IFNγ [67]) plays a dominant role in viraemia suppression in these mice (Fig. 3B). For CHIKV to evolve a capacity to counter the anti-viral effects of TNF may be unrealistic in the limited time frame.

Deep sequencing of virus isolated day 100 from Rag1^−/−^ mouse serum showed only a limited number of mutations (S1 Figure B in S1 Text) and a limited quasi-species diversity (Fig. S1C in Text S1); perhaps surprising given the low fidelity of viral RNA replication [68]. Alphavirus isolation generally involves virus expansion *in vitro* (in this case using C6/36 cells), which may bias the results [69]; however, many genetically diverse alphaviruses can be expanded on C6/36 cells. These results (S1 Figure B, C, in S1 Text) would therefore suggest that despite 100 days of continuous replication in Rag1^−/−^ mice, a highly diverse infectious virion quasi-species population was not generated [70].

### Foot swelling and arthritis

**Figure 4:**
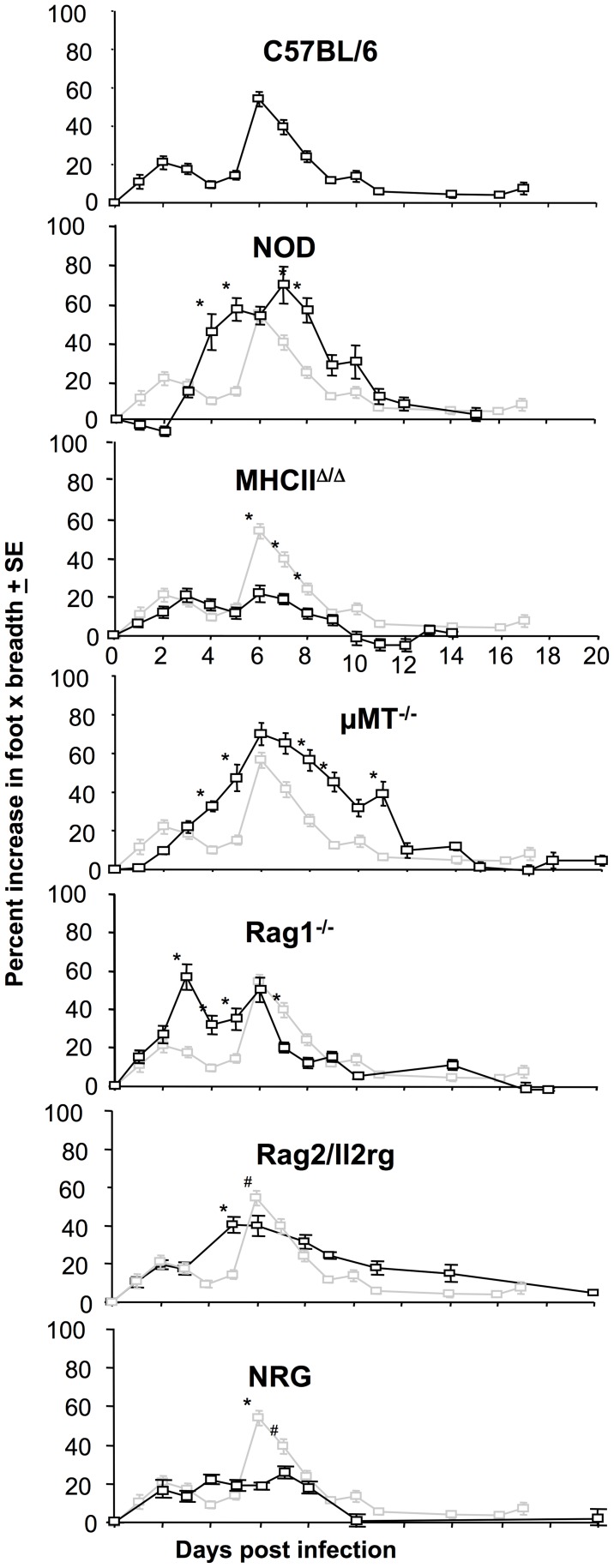
Foot swelling in different mouse strains. Foot swelling was determined in the same mouse strains shown in Fig. 1. C57BL6 (n = 18–28 feet, data from 6 independent experiments), NOD (n = 8), MHCII^Δ/Δ^ (n = 14–18, 2 independent experiments), µMT (n = 16–36, 4 independent experiments), Rag1^−/−^ (n = 12–26, 3 independent experiments), Rag2/Il2rg (n = 8), NRG (n = 10). Significance by Mann Whitney U or Kolmogorov-Smirnov tests * p<0.008, # p<0.04. The light graphs show foot swelling in C57BL/6 mice for comparison.

The mouse strains shown in Fig. 1 were also analyzed for foot swelling post CHIKV infection. Relative to C57BL/6 mice, NOD mice showed a clear increase in foot swelling (Fig. 4, NOD). Foot swelling in NOD mice was associated with profound cellular infiltrates and edema (S2 Figure A in S1 Text). NOD mice have a range of immune defects that could contribute to exacerbated CHIKV arthritis (see Discussion).

MHC II^Δ/Δ^ and NRG mice show clearly reduced foot swelling when compared with C57BL/6 mice (Fig. 4, C57BL/6, MHC II^Δ/Δ^ and NRG), consistent with previous data showing that CD4 T cells are important for driving CHIKV arthritis [19], [20]. Curiously, Rag1^−/−^ mice (also T cell deficient) showed no reduction in foot swelling compared with C57BL/6 mice. However, histological examination illustrated that this swelling in Rag1^−/−^ mice was largely due to edema, both on day 3 and day 6, with the density of cellular infiltrates actually lower in Rag1^−/−^ mice than in C57BL/6 mice (S2 Figure B in S1 Text); an observation consistent with previous findings [36]. T cells would thus appear to be involved in the marked recruitment of inflammatory cells that characterizes arthritic disease in C57BL/6 mice [51].

Rag2/Il2rg mice showed less foot swelling than Rag1^−/−^ mice on day 3 (Fig. 4, Rag2/Il2rg vs Rag1^−/−^), perhaps suggesting a role for NK cells in promoting edema (a contention proposed previously [71]). CHIKV infections are well known to induce edema [72].

Foot swelling was significantly higher in µMT mice than C57BL/6 mice on days 4, 5, 7, 8, 9 and 11 (Fig. 4, µMT), consistent with a previous report using a different CHIKV isolate [37]. The density of the cellular infiltrates was similar in µMT and C57BL/6 mice (S2 Figure in S1 Text), illustrating that the foot swelling in µMT mice was not simply due to edema. The increased arthritis from day 4 onwards in µMT mice (which have T cells) is consistent with the arthritogenic role of CHIKV-specific CD4 T cells [19], [20]. Loss of viraemia control in B cell-deficient mice (including µMT mice) significantly diverged from C57BL/6 mice on day 4 post-infection (Fig. S3A in Text S1), consistent with the appearance of neutralizing antibodies on day 4 post-infection in C57BL/6 mice (S3 Figure B in S1 Text). The increased viraemia from day 4 onwards in µMT mice presumably leads to the exacerbated arthritic disease.

Despite the reported roles of Fc receptors in suppressing antiviral responses and promoting arboviral disease [73], [74], [75], foot swelling and viraemia was largely unaffected in mice deficient for the common gamma chain of the Fc receptor (FcRγ) (S4 Figure in S1 Text).

### Pathology associated with persistent viraema in Rag1^−/−^ and NRG mice

The persistent viraemias in the B cell deficient mouse strains (with the exception of NRG mice - see below) resulted in surprisingly little overt pathology with mice appearing and behaving normally based on regular monitoring by trained animal house staff. To investigate further the pathological effects of a persistent alphaviral infection, the liver, lungs, brain, spleen, lymph nodes, muscle, skin, and feet of Rag1^−/−^ mice chronically infected for 430 days were examined by histology. The only clear histopathological modifications associated with infection were a marked increase in granulocytosis and granulopoiesis in the spleen (S5 Fig in S1 Text), a feature previously associated with infection and inflammation [76]. Persistent alphaviral replication in Rag1^−/−^ mice was thus associated with surprisingly little pathology identifiable by standard histology. A previous study using young Rag1^−/−^ mice, showed persistent infection and mild persistent joint pathology [36], perhaps consistent with increased disease associated with alphavirus infection of young mice [10], [77], [78].

In NRG mice the chronic CHIKV viraemia was eventually associated with morbidity and mortality. NRG mice often showed altered gait and balance, and impaired hind foot limb movement, with animals requiring euthanasia between days 120 and 230 (S6 Figure in S1 Text). These signs and symptoms are suggestive of neurological disease [79], [80], [81]. Histological examination of brain tissue from euthanized mice showed clear signs of on-going inflammation in the central nervous system, with severe vacuolization and edema, astrocytosis, microgliosis, and mild degeneration of neurons evident (S7 Figure A in S1 Text). Immunohistochemistry with an anti-capsid antibody showed that both neurons and oligodendrocytes were infected (S7 Fig B in S1 Text). Infection of these cells has been reported previously for the encephalopathies caused by CHIKV [82] and other alphaviruses [81].

### Persistent CHIKV RNA and negative-strand CHIKV RNA in C57BL/6 mice

**Figure 5:**
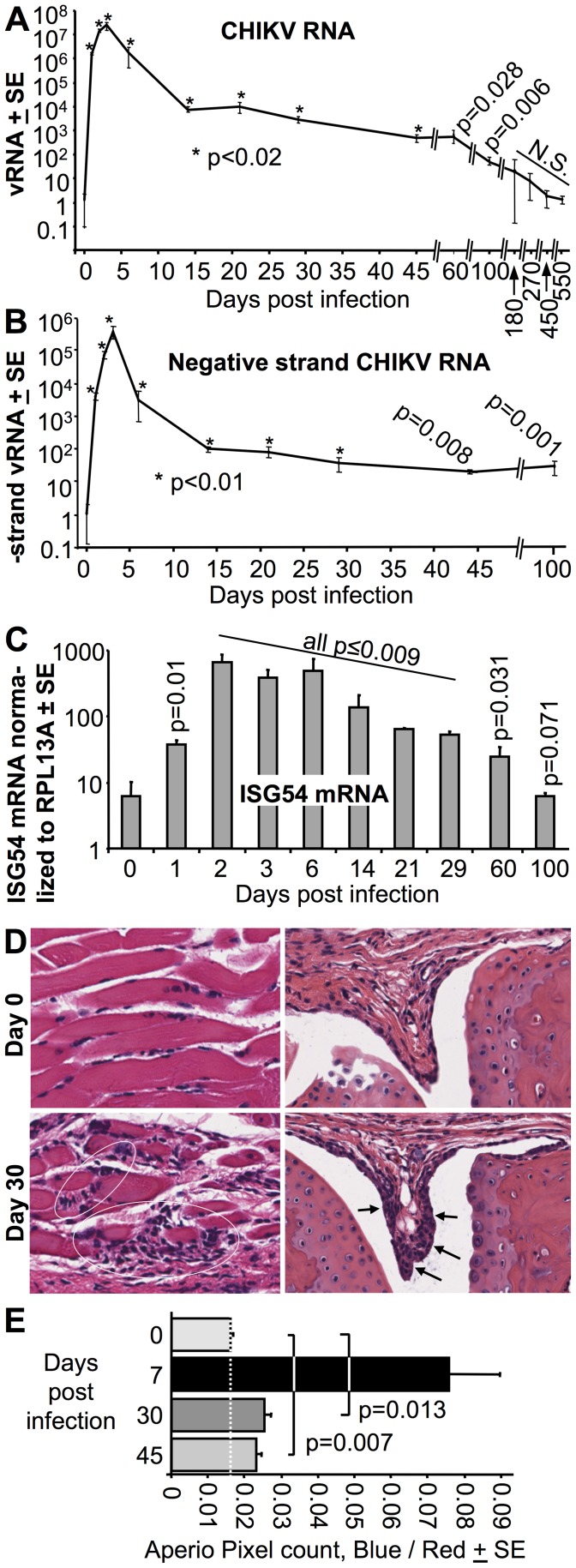
Persistent CHIKV RNA, up-regulation of ISG54 and inflammatory infiltrates in C57BL/6 mice. (A–C) RNA was isolated from feet of infected mice at the indicated time points and analyzed by qRT-PCR. (A) CHIKV RNA levels determined by standard qRT PCR using primers specific for E1 normalized to RPL13A mRNA levels (n = 3–7 feet from independent mice per time point). Statistics by Mann-Whitney U tests comparing RNA levels at the indicated times post infection with levels at time 0. (B) Negative strand-specific qRT PCR using primers specific for nsP1 (n and statistics as for A). (C) ISG54 RNA levels as determined by standard qRT PCR, normalized to RPL13A mRNA levels (n and statistics as for A). (D) H & E staining of feet day 0 and day 30 post CHIKV infection. Infiltrates are shown for day 30 in muscle tissue (bottom left, while circles) and in the synovial membrane (bottom right, black arrows). (E) Aperio Positive Pixel Count determination of the ratio of blue (nuclear) to red (cytoplasmic) staining areas in whole foot sections at the indicated times post-infection. Leukocytes tend to have a high nuclear/cytoplasmic area ratio, so elevated ratios in whole foot sections indicate the presence of leukocyte infiltrates. (n = 3/4 feet from independent mice per time point, statistics by t test).

A number of reports have suggested that virus and/or viral RNA of CHIKV and other alphaviruses persists *in vivo* long after the viraemia has abated [20], [36], [38], [40], [66], [83]. Persistence of CHIKV RNA was also seen in our C57BL/6 mouse model, with significant levels of CHIKV RNA detected by standard qRT PCR for 100 days post infection in feet (Fig. 5A); this method measures the levels of both positive-strand and negative-strand CHIKV RNA. Significant levels of negative-strand RNA, detected by strand-specific qRT PCR [50], were also seen over the same period (Fig. 5B). qRT-PCR analyses of spleens revealed that no significant levels of CHIKV RNA were detectable from day 14 (S8 Fig A in S1 Text), consistent with a published report using CHIKV infection of young mice [84].

The time course of viral RNA and negative-stranded RNA levels in feet showed a rapid decline from the peak viraemia (day 3) to the end of the viraemic period (day 6) (Fig. 5A, B; Fig. S8B in Text S1), a drop likely largely mediated by anti-CHIKV antibodies inhibiting viral infection (S3 Figure A, B, in S1 Text). Thereafter CHIKV RNA levels in the feet fell more slowly (Fig. 5A, B; S8 Figure B in S1 Text), with curve fitting from day 14 onwards suggestive of an exponential decay with a half life of ≈10–11 days for both RNA and negative-strand RNA levels (S8 Figure B in S1 Text).

### Persistent ISG54 up-regulation, CHIKV antigen and arthritic inflammation in C57BL/6 mice

Significant levels of mRNA of the IFNα/β stimulated gene, ISG54 [85], were detected for up to 60 days post infection (Fig. 5C), suggesting ongoing stimulation of IFNα/β responses by persistent CHIKV RNA. Immunohistochemistry using a new monoclonal antibody that recognizes the capsid protein of CHIKV (Goh et al. submitted), also indicated the presence of capsid-positive cells in foot tissues on day 30 post infection (S9 Figure in S1 Text), suggesting the persistent RNA is translationally active. Although foot swelling was no longer detectable after day 10–12 in C57BL/6 mice, histological examination of feet illustrated the presence of small foci of inflammatory infiltrates; examples of such lesions in muscle and synovial membrane are shown (Fig. 5D). Quantitation using Aperio Positive Pixel Count analyses of whole foot sections confirmed that significantly elevated levels of these infiltrates could be detected up to day 45 post infection (Fig. 5E).

This mouse model of CHIKV infection thus recapitulates the persistence of viral RNA and protein seen in monkeys and humans [38], [40], and supports the view that such persistence gives rise to chronic inflammatory arthropathy [39], [86].

### Transcriptomic analysis C57BL/6 feet at day 30 post infection

To gain insights into the chronic inflammatory signature in C57BL/6 mice, a microarray analysis was undertaken as described previously [19] using feet from C57BL/6 mice at day 0 and 30 post-infection; (a principal component analysis is shown in S10 Figure A in S1 Text). The fold changes in gene expression on day 30 (relative to day 0) were generally much lower (range 1.44–12.21 fold, S3 Table in S1 Text) than the changes seen during peak disease (day 7) [19], likely due to the >2 logs lower levels of CHIKV in the feet at this time (Fig. 5A, B). Nevertheless, 192 significantly up-regulated genes were identified (S3 Table S3 in S1 Text); (a heat map of these genes is shown in S10 Figure B in S1 Text). Differential expression of two genes (in addition to ISG54, Fig. 5C) was also demonstrated by qRT-PCR (S10 Figure C in S1 Text). Ingenuity Pathway Analysis of the 192 genes suggested activation of canonical pathways associated with T cells, autoimmunity, antigen presentation, NK cells, innate sensing (primarily IFNα/β pathways), monocytes/macrophages, apoptosis and cytokines (S11 Figure in S1 Text). These pathway groupings were broadly similar to those described for day 7 post infection [19], suggesting acute and chronic arthritis share many inflammatory processes.

The same 192 up-regulated genes were analyzed using Ingenuity Pathway Analysis of upstream regulators (Table 1, S4 Table in S1 Text). This analysis provided evidence for stimulation of pathways involved in type I IFN responses and supports the view that persistent CHIKV RNA continues to stimulate these responses; it is also consistent with Fig. 5C and the up-regulation of IFNα in joints of chronic CHIKV patients [40]. Poly(ADP-ribose) polymerase-1 (PARP-1) and IL-6 were identified as upstream regulators (Table 1), with these also up-regulated in the synovial tissues of a chronic CHIKV patient [40]. Cleavage of PARP-1 is associated with CHIKV-induced apoptosis [87] and up-regulation of serum IL-6 has been associated with chronic CHIKV disease [88]. Upstream regulators associated with T cells and Th1 responses (IFNγ and IL-12) were identified (Table 1), with such responses again seen in chronic CHIKV patients [40], [46]. These results suggest that the mouse model used herein recapitulates many of the chronic inflammatory pathways seen in humans. STAT3 was also identified (Table 1), with this transcription factor associated with M2 macrophage differentiation [89], [90]; M2 differentiation was recently shown to be associated with CHIKV persistence [91]. IRGM (Table 1) is an autophagy-associated protein targeted by CHIKV-NS2 and E3 proteins [92], with viruses believed to manipulate autophagy to promote their own replication [93].

**Table 1:** Upstream regulator analysis of 192 up-regulated genes from mouse feet at day 30 post CHIKV infection compared to day 0.

Upstream Regulator	Activation z-score	p-value of overlap
**Type I IFN responses**		
Ifnar	4.5	1.4E-27
IFNB1	3.8	1.4E-23
IRF7	4.3	5.9E-20
STAT1	4.3	2.4E-17
IRF3	3.6	7.4E-16
TLR3	3.6	2.4E-14
Interferon alpha	4.4	5.1E-14
IFNL1	3.4	7.5E-14
IFNAR1	2.8	3.6E-13
IFN Beta	3.4	6.5E-13
IFNA2	3.7	2.0E-12
IFN alpha/beta	3.3	5.6E-12
IFNA1/IFNA13	2.9	8.5E-12
TLR4	3.3	1.3E-11
IFN type 1	2.9	2.2E-11
DDX58 (RIG-I)	2.0	4.0E-11
IFNE	2.4	8.3E-11
IFNK	2.2	1.1E-10
IKBKE	0.1	9.7E-10
**Negative regulators of type I IFN responses**		
SOCS1	−3.9	4.6E-20
TRIM24	−4.1	9.4E-20
mir-21	−3.8	1.0E-17
**Cytokines**		
IFNγ (Th1)	6.3	1.1E-21
IL12 (complex) (Th1)	1.8	5.7E-11
IL6	3.9	6.7E-10
**T cell responses**		
DOCK8	3.2	3.6E-11
TCR	0.5	5.3E-11
**Apoptosis**		
PARP1	3.1	1.6E-10
**M2 differentiation**		
STAT3	2.3	2.5E-10
**Autophagy**		
IRGM	−2.8	3.8E-10
**Others**		
PLK4	2.6	2.2E-10
PLK2	2.6	3.0E-10
FZD9	2.4	9.2E-10

All upstream regulators with a p value <10e-9 are listed. A positive z-score indicates that the identified regulator is activated, since it normally up-regulates specific genes found in the 192 gene set. Similarly, a negative z-score indicates that the identified regulator is inhibited, since it normally down-regulates specific genes found in the 192 gene set. The full data set from this analysis with associated lists of up-regulated genes is shown in S4 Table in S1 Text.

## Discussion

Overall this paper highlights the propensity for CHIKV to persist *in vivo* both as a persistent steady-state viraemia in B cell deficient mice and as persistent RNA in C57BL/6 mice. The study also provides evidence that other factors aside from antibodies and IFNα/β, primarily CD4 T cells, are active in alphaviral viraemia suppression. Finally the limited pathology associated with persistent viraemias (except when levels were very high – see below), and the lack of correlation between viraemia and arthritic disease, underscores the immunopathological basis of CHIKV arthropathy.

### Immune factors involved in viraemia suppression

Herein we show that mice deficient in B cells maintain persistent, relatively stable “set point” viraemias reminiscent of those seen in HIV patients [52]. These experiments suggest that T cells play a role (albeit secondary to IFNα/β and antibodies) in suppressing viraemia, with the set-point viraemia ≈2 logs higher in Rag1^−/−^ mice (B and T cell deficient) than in µMT mice (B cell deficient). Vaccination studies in µMT mice further support a role for T cells in CHIKV viraemia suppression (Fig. 2 B, C). CD4 T cells (rather than CD8 T cells) are implicated in this anti-viral activity [20], [32], [59], with direct antiviral roles for CD4 T cells also described for other viruses [94], including encephalitic alphaviruses [95].

Cytokine analysis in Rag1^−/−^ mice showed persistently elevated levels of circulating IFNγ and TNF, with both of these cytokines known to have anti-alphaviral activity [63], [64]. Although neutralization of TNF has been shown to be lethal in the related Ross River virus mouse model [64], CHIKV infection of IFNγ ^−/−^ mice did not show any significant increases in viraemia when compared with C57BL/6 mice in this mouse model [19], (although slight increases were observed by others using a slight different model and assay system [20]). As both antibody [96] and IFNα/β responses remain active in IFNγ^−/−^ mice, the contribution of IFNγ may be hard to dissect in this setting.

The ≈0.8 log higher set point viraemia in T, B and NK deficient mice with a NOD background compared with T, B and NK deficient mice on a C57BL/6 background (Fig. 1, NRG vs. Rag2/Il2rg), suggests that further innate factors are involved in viraemia suppression. The NOD background has a number of defects in innate immunity that might be involved including (i) altered apoptosis [54], IFNγ signaling [53] and/or IL-1β production [97], [98] in macrophages, (ii) NKT cell deficiencies [99], and/or (iii) lack of C5 complement activity [55], [100]. Given that NK cells do not appear to be involved in viraemia control, the NK defects in NOD mice [101] are presumably not involved.

### Correlates of arthritic pathology

Viraemia levels were not a good predictor of arthritic disease, consistent with human studies [102]. Instead, arthritic disease was associated with the presence of T cells, consistent with the arthritogenic role of CD4 T cells in CHIKV infections [19], [20]. Vaccination experiments in µMT mice also highlighted a potential problem if vaccines were to induce CD4 T cell responses, but inadequate B cell responses, with such a scenario resulting in exacerbated arthritic disease post infection (Fig. 2D). T cell driven pathology may also contribute to the severe disease seen in neonates born to CHIKV infected mothers (see references in [1]), as maternal antibodies are well known to inhibit the offspring's own antibody production, whilst allowing generation of T cell responses [103].

The increase in arthritis seen in NOD mice (that have intact T, B and NK cells) when compared with C57BL/6 mice (Fig. 4) may involve the NOD defects listed above and/or other defects [104], [105], although complement defects might be expected to ameliorate disease [100], [106]. Increased viral replication (as suggested by increased viraemia in NRG mice - Fig. 1) in some key cell types may also play a role in exacerbating arthritis. A role for autoimmune T cells is improbable as there is no evidence that such cells are responsible for alphaviral arthritides [39], with self-reactive diabetogenic T cells in NOD mice restricted to a subset of T cells that recognize a specific insulin epitope [107].

### Encephalitic pathology

The mouse strains lacking B cell responses developed surprisingly little pathology, despite the persistent viraemias, suggesting that the cytopathic alphaviral infections, in themselves, are generally not major drivers of disease. However, this contention likely does not hold true in NRG mice, which show relatively higher levels of persistent viraemia and ultimately develop encephalitis, with CHIKV infection of neurons and oligodendrocytes evident. Infection and killing of neurons is believed to be responsible for encephalopathy in the Sindbis virus mouse model [80] and may also play a role in CHIKV encephalitis [82], [108], [109]. In contrast to the alphaviral encephalitis induced by Semliki Forest virus [81], conventional T cells are unlikely to be involved in NRG mice (as these mice are defective for Rag1 activity). The high viraemias in NRG mice may promote the encephalopathy, as high CHIKV viraemias have been associated with lethal encephalitis (i) in mice deficient for the IFNα/β receptor [10] and (ii) in monkeys inoculated with high levels of CHIKV [38]. However, CHIKV encephalitis in humans and primates generally occurs during acute disease and near the peak viraemia [38], [110], [111], rather than eventually arising from an extended viraemia.

### Persistent CHIKV RNA

The C57BL/6 adult mouse model used herein recapitulates (i) the persistence of CHIKV RNA and protein seen in humans and monkeys and (ii) many of the persistent inflammatory responses seen in humans with chronic CHIKV arthropathy. This mouse model might thus be viewed as a model of both acute [48] and chronic CHIKV disease.

The nature of persistent CHIKV RNA and protein remains poorly understood. A key question is whether such persistence simply represents material left over after active replication of virus in tissues, or alternatively, involves ongoing replication of virus or viral RNA [67], [112]. Notwithstanding the propensity of alphaviruses to maintain persistent infections *in vivo* (Fig. 1) and the aforementioned human and monkey studies [38], [40], several lines of evidence presented herein support the view that the persistent CHIKV RNA is replicating: (i) the relatively long, 10–11 day half-life of CHIKV RNA (S8 Figure B in S1 Text) compared with the reported 3–10 hour half-life of cellular Sindbis virus RNA [113], [114]; (ii) the presence of CHIKV negative-strand RNA [50] (packaged virions only containing positive-strand RNA [115]); (iii) ongoing induction of double-stranded RNA, TLR3 and IFNα/β-inducible ISG54 [85], [116] and (iv) the ability to detect CHIKV structural proteins on day 30 post-infection, with capsid synthesis requiring generation of subgenomic positive-strand RNA (from negative strand RNA) by viral RNA-dependent RNA polymerase [115]. Although CHIKV RNA appears to persist, we have been unable to isolate infectious virus from C57BL/6 mice beyond day 14 using a number of sensitive techniques (S5 Table in S1 Text). This observation is consistent with the inability to isolate infectious virus from patients with chronic alphaviral disease, despite the presence of persistent alphaviral RNA [40], [117], [118].

The microarray study suggests chronic inflammatory disease is similar in mice and humans [40], [46], [88], with IFNα/β, T cells, IL-12, IFNγ and IL-6 continuing to be stimulated long after the end of the viraemic period. Such responses are likely involved in ongoing arthritic inflammation and chronic disease [40], [88]. However, whether they also ultimately help to clear persistent CHIKV RNA/protein is unclear; clearance of persistent Sindbis virus from neurons is thought to involve antibodies and T cell IFNγ [47]. Aged monkeys with reduced T cell responses also showed increased viral persistence [119]; however, T cell responses were not different in recovered compared with chronic CHIKV patients [46]. Persistent CHIKV RNA is believed to reside in macrophages [38], [40], with tissue-resident rather than inflammation-recruited macrophages recently implicated [51]. Perhaps noteworthy is that the estimated ≈10-11 day half-life of persistent RNA (Fig. S8B in Text S1) is nominally remarkably close to the natural turnover rate of tissue macrophages, estimated to be 21–27 days for total replacement [120], [121]. Further work is clearly needed to understand how viral RNA persists, and to differentiate between those immune responses required for viral clearance and those driving chronic arthropathy.

## Supporting Information

Text S1 This file contains supporting information tables and figures. **Table S1.** The effect of age of mice within a 6–12 week old age range on peak foot swelling. **Table S2.** List of all the mice strains used in the current study. **Table S3.** Table of 192 genes identified by microarray as up-regulated in feet on day 30 post infection. **Table S4.** Table showing the full data set for upstream regulators shown in Table 1. **Table S5.** Attempts to isolate replication competent virus from foot tissues of CHIKV infected C57BL/6 mice≥30 days post infection. **Figure S1 A.** Foot swelling and/or viraemia in C57BL/6 mice infected with CHIKV isolates recovered from Rag1^−/−^ mice days 100 and 429 post infection. **Figure S1 B.** Mutations identified in CHIKV recovered from Rag1^−/−^ mice day 100 post infection. **Figure S1 C.** IGV display of deep sequencing output for parental and Rag100 viruses. **Figure S2** **A.** H&E staining of feet after CHIKV infection of NOD mice showing inflammatory infiltrates in synovial membrane, muscle and dermis. **Figure S2 B.** Quantitation of cellular infiltrates in C57BL/6, Rag1^−/−^, µMT, and MHCII ^Δ/Δ^ mice. **Figure S3.** Early loss of viraemia control in B cell deficient mice, and neutralising antibody responses in C57BL/6 mice. **Figure S4.** Viraemia and foot swelling in FcγR^−/−^ mice. **Figure S5.** Histopathological modifications in spleens of chronically CHIKV infected Rag1^−/−^ mice. **Figure S6.** Survival of NRG mice post CHIKV infection. **Figure S7 A.** Brain lesions in NRG mice with neurological symptoms requiring euthanasia. **Figure S7 B.** Immunohistochemical staining of CHIKV capsid protein in brain tissue of NRG mice with neurological symptoms requiring euthanasia. **Figure S8 A.** No persistent CHIKV RNA in spleen. **Figure S8 B.** Curve fitting of decline in persistent post-viraemic CHIKV RNA in wild-type mouse feet over time. **FigureS9.** Persistence of viral antigen in feet of CHIKV infected mice. **Figure S10 A.** Principal component analysis of 4,805 filtered genes identified by microarray analysis of feet of wild-type mice day 0 and 30 post infection. **Figure S10 B.** Heat map of 192 significantly up-regulated genes identified by microarray analysis of feet of wild-type mice day 30 post infection. **Figure S10 C.** qRT PCR of granzyme B and FcγR4 at days 0, 7 and 30 post infection with CHIKV. **Figure S11.** Ingenuity Pathway Analysis showing canonical pathways associated with the 192 genes up-regulated in feet of mice at day 30 post infection.
(PDF)
