# Supplementary material for: Multiple Immune Factors Are Involved in Controlling Acute and Chronic Chikungunya Virus Infection
Source: PLoS Negl Trop Dis. 2014 Dec 4;8(12):e3354. doi: 10.1371/journal.pntd.0003354 (PMC4256279; doi:10.1371/journal.pntd.0003354)

Table S1. The effect of age of mice within a 6-12 week old age range on peak foot swelling day 6. None of the groups were significantly different. The same virus batch was used for all experiments.

**Age or age range, weeks**	**n (feet)**	**Day 6 % increase in foot swelling ± SE**
6	8	53.3 ± 8.1
6.5	6	46.5 ± 12.4
6 - 10	8	50 ± 6.9
8 - 12	10	48 ± 4.9
11 - 12	10	59.6 ± 16.4

Table S2. List of all the mice strains used in the current study

Mice strain	Nomenclature	Description
C57BL/6	C57BL/6	Wild-type C57BL/6 mice
NOD	NOD/ShiLtJ	Non-obese diabetic mouse produced by inbreeding of Cataract Shinogi strain.
MHC-II$^{\Delta/\Delta}$	B6.129S2-*H2*$^{dlAb1\text{-}Ea}$/J	MHC II deficient C57/BL6 mice designed via targeted deletion of 78.8 kb MHC class II genes (*H2-Ab1, H2-Aa, H2-Eb1, H2-Eb2, H2-Ea*).
µMT	B6.129S2-*Igh-6*^{tm1Cgn}/J	B cells deficient C57/B6 mice designed via targeted disruption of the membrane exon of the immunoglobulin µ chain gene.
Rag-1$^{-/-}$	B6.129S7-*Rag1*^{tm1Mom}/J	B and T cells deficient C57/BL6 mice designed via targeted mutation of *Rag1*^{tm1Mom}.
Rag2/Il2rg	B6.*Rag2*^{tm1Fwa}*Il2rg*^{tm1Wjl}	B, T and NK cells deficient C57/BL6 mice designed by crossing Rag2$^{-/-}$ mice and *Il2rg*$^{-/-}$ mice.
NRG	NOD.Cg-*Rag1*^{tm1Mom} *Il2rg*^{tm1Wjl}/SzJ	B, T and NK cell deficient NOD-congenic mice designed via targeted mutation of *Rag1*^{null} mutation and the *IL2rγ*^{null} mutation.
FcγR$^{-/-}$	B6.129P2-*Fcer1gtm1Rav* N12	FcγRIII and FcεRI deficient C57/BL6 mice designed via targeted mutation of *Fcer1g*^{tm1Rav}

Table S3

probeset_id	Probeset Type (mouse)	Symbol (mouse)	Description (mouse)	Synonyms (mouse)	Type of gene (mouse)	DAY 0	DAY 0	DAY 30	DAY 30	d30_vs_d0 (p-value)	Mean d0	Mean d30	log2fold-change	fold change
10531407	GeneSymbol	Cxcl9	chemokine (C-X-C motif) ligand 9	BB139920|CMK|Mig|Scyb9|crg-10	protein-coding	6.55	6.65	10.47	9.95	0.0053	6.6	10.21	3.61	12.21
10455961	GeneSymbol	Iigp1	interferon inducible GTPase 1	2900074L10Rik|AI046432|AW111922|Iigp|Irga6	protein-coding	5.49	5.39	8.49	7.85	0.0138	5.44	8.17	2.73	6.63
10385518	GeneAccession	Tgtp1	T-cell specific GTPase 1	Gtp2|Irgb6|Mg21|Tgtp	protein-coding	6.11	6.32	8.93	8.56	0.0070	6.215	8.745	2.53	5.78
10496592	GeneSymbol	Gbp2	guanylate binding protein 2	-	protein-coding	6.83	6.39	9.82	8.76	0.0429	6.61	9.29	2.68	6.41
10385533	GeneAccession	Tgtp1	T-cell specific GTPase 1	Gtp2|Irgb6|Mg21|Tgtp	protein-coding	6.01	6.34	8.96	8.3	0.0218	6.175	8.63	2.455	5.48
10531987	GeneSymbol	Gbp4	guanylate binding protein 4	AW228052|KIAA4245|Mag-2|Mpa-2|Mpa2|mKIAA4245	protein-coding	6.32	6.3	9.11	8.34	0.0245	6.31	8.725	2.415	5.33
10455954	GeneSymbol	Gm4951	predicted gene 4951	EG240327	protein-coding	5.48	5.37	7.59	6.93	0.0317	5.425	7.26	1.835	3.57
10590635	GeneSymbol	Ccr5	chemokine (C-C motif) receptor 5	AM4-7|CD195|Cmkbr5	protein-coding	5.62	5.57	7.25	7.37	0.0014	5.595	7.31	1.715	3.28
10598013	GeneSymbol	Ccr5	chemokine (C-C motif) receptor 5	AM4-7|CD195|Cmkbr5	protein-coding	5.62	5.57	7.25	7.37	0.0014	5.595	7.31	1.715	3.28
10351509	GeneSymbol	Fcgr4	Fc receptor, IgG, low affinity IV	4833442P21Rik|CD16-2|FcgRIV|FcgammaRIV|Fcgr3a|Fcrl3	protein-coding	5.25	5.34	7.01	6.81	0.0046	5.295	6.91	1.615	3.06
10559486	GeneSymbol	Lair1	leukocyte-associated Ig-like receptor 1	5133400O11Rik|BB115266|D7Bwg0421e|Lair-1|mLair-1	protein-coding	6.15	5.93	7.81	7.71	0.0049	6.04	7.76	1.72	3.29
10542164	GeneSymbol	Clec12a	C-type lectin domain family 12, member a	CLL-1|D230024O04|Micl	protein-coding	6.66	6.54	8.45	8.49	0.0011	6.6	8.47	1.87	3.66
10354732	GeneSymbol	Hspd1	heat shock protein 1 (chaperonin)	60kDa|Hsp60	protein-coding	7.21	6.48	8.99	8.51	0.0488	6.845	8.75	1.905	3.75
10496580	GeneSymbol	Gbp3	guanylate binding protein 3	AW228655|Gbp4	protein-coding	6.34	6.22	8.28	7.73	0.0256	6.28	8.005	1.725	3.31
10531994	GeneSymbol	Mpa2l	macrophage activation 2 like	AI595338|GBP6	protein-coding	7.13	6.69	8.97	8.6	0.0227	6.91	8.785	1.875	3.67
10416837	GeneSymbol	Irg1	immunoresponsive gene 1	AI323667	protein-coding	6.07	6.58	8.18	7.72	0.0419	6.325	7.95	1.625	3.08
10462632	GeneSymbol	Kif20b	kinesin family member 20B	B130024C23|C330014J10Rik|Mphosph1	protein-coding	5.46	5.24	6.83	6.59	0.0140	5.35	6.71	1.36	2.57
10375515	GeneSymbol	Ifi47	interferon gamma inducible protein 47	47kDa|IRG-47|Igrd|Iigp4|Iipg4|Irgd|Olfr56	protein-coding	5.46	5.26	6.76	6.59	0.0098	5.36	6.675	1.315	2.49
10531415	GeneSymbol	Cxcl10	chemokine (C-X-C motif) ligand 10	C7|CRG-2|INP10|IP-10|IP10|Ifi10|Scyb10|gIP-10|mob-1	protein-coding	6.87	7.02	8.23	8.91	0.0430	6.945	8.57	1.625	3.08
10608646	Transcribed Locus	NA	NA	NA	Unknown	6.2	6.33	7.99	7.44	0.0359	6.265	7.715	1.45	2.73
10376326	GeneSymbol	Irgm2	immunity-related GTPase family M member 2	AI481100|GTPI|Iigp2|MGC102455	protein-coding	6.8	6.78	8.44	8.23	0.0046	6.79	8.335	1.545	2.92
10538590	GeneSymbol	Herc5	hect domain and RLD 5	1700121D12Rik|2510038N07Rik|4930427L17Rik|AI451296|CEB1|Herc6|KIAA0032	protein-coding	5.92	5.63	7.25	6.92	0.0270	5.775	7.085	1.31	2.48
10496569	GeneSymbol	Gbp6	guanylate binding protein 6	9830147J24Rik|Gbp7	protein-coding	7.1	6.81	8.44	8.21	0.0178	6.955	8.325	1.37	2.58
10406928	GeneSymbol	Cd180	CD180 antigen	F630107B15|Ly78|RP105	protein-coding	7.61	7.45	9	9.02	0.0030	7.53	9.01	1.48	2.79
10566583	GeneSymbol	Gm8995	predicted gene 8995	A630026L20|EG668139	unknown	7.39	7.15	8.78	8.59	0.0115	7.27	8.685	1.415	2.67
10461721	GeneSymbol	Mpeg1	macrophage expressed gene 1	MGC150002|MGC150003|MPS1|Mpg-1	protein-coding	7.09	7.13	8.36	8.62	0.0090	7.11	8.49	1.38	2.60
10590631	GeneSymbol	Ccr2	chemokine (C-C motif) receptor 2	Cc-ckr-2|Ccr2a|Ccr2b|Ckr2|Ckr2a|Ckr2b|Cmkbr2|mJe-r	protein-coding	6.2	6.01	7.25	7.31	0.0071	6.105	7.28	1.175	2.26
10385500	GeneSymbol	Irgm1	immunity-related GTPase family M member 1	Ifi1|Iigp3|Iipg3|Irgm|LRG-47	protein-coding	7.14	6.64	8.36	8.07	0.0444	6.89	8.215	1.325	2.51
10566358	GeneSymbol	Trim30	tripartite motif-containing 30	Rpt-1|Rpt1	protein-coding	6.87	6.73	8.02	8.15	0.0055	6.8	8.085	1.285	2.44
10531972	GeneAccession	Gbp8	guanylate-binding protein 8	5830443L24Rik|mGBP8	protein-coding	5.93	6.02	7.19	6.99	0.0095	5.975	7.09	1.115	2.17
10360173	GeneSymbol	Slamf7	SLAM family member 7	19A|19A24|4930560D03Rik|CRACC|CS1	protein-coding	6.45	6.57	7.89	7.55	0.0215	6.51	7.72	1.21	2.31
10566585	GeneSymbol	Gm1966	predicted gene 1966	-	protein-coding	6.64	6.45	7.51	7.99	0.0429	6.545	7.75	1.205	2.31
10466190	GeneSymbol	Ms4a14	membrane-spanning 4-domains, subfamily A, member 14	Gm1276	protein-coding	5.9	5.58	6.65	6.93	0.0386	5.74	6.79	1.05	2.07
10453715	Transcribed Locus	NA	NA	NA	Unknown	6.37	6.05	7.49	7.2	0.0343	6.21	7.345	1.135	2.20
10358408	GeneSymbol	Rgs1	regulator of G-protein signaling 1	BL34	protein-coding	5.83	5.51	6.7	6.69	0.0235	5.67	6.695	1.025	2.03
10531980	GeneAccession	Gbp9	guanylate-binding protein 9	E430029F06	protein-coding	6.36	6.32	7.67	7.23	0.0374	6.34	7.45	1.11	2.16
10350392	GeneSymbol	Aspm	asp (abnormal spindle)-like, microcephaly associated (Drosophila)	Calmbp1|D330028K02Rik|FLJ10518|MCPH5|Sha1	protein-coding	6.59	6.63	8	7.52	0.0412	6.61	7.76	1.15	2.22
10376324	GeneSymbol	Gm12250	predicted gene 12250	Irgb10|OTTMUSG00000005723	protein-coding	5.81	6.02	6.95	6.93	0.0104	5.915	6.94	1.025	2.03
10404606	GeneSymbol	Ly86	lymphocyte antigen 86	MD-1|MD1	protein-coding	8.54	8.1	9.68	9.81	0.0249	8.32	9.745	1.425	2.69
10519713	GeneSymbol	LOC635773	hypothetical protein LOC635773	-	pseudo	5.51	5.67	6.65	6.43	0.0199	5.59	6.54	0.95	1.93
10566574	GeneSymbol	Gvin1	GTPase, very large interferon inducible 1	9130002C22Rik|9830104F22Rik|Iigs1|VLIG|VLIG-1	protein-coding	6.86	7.04	8.28	7.97	0.0225	6.95	8.125	1.175	2.26
10571984	GeneSymbol	Ddx60	DEAD (Asp-Glu-Ala-Asp) box polypeptide 60	9830118M07|MGC19297	protein-coding	5.94	5.56	6.67	6.77	0.0387	5.75	6.72	0.97	1.96
10422760	GeneSymbol	Fyb	FYN binding protein	ADAP|B630013F22Rik	protein-coding	6.58	6.36	7.54	7.55	0.0103	6.47	7.545	1.075	2.11
10462623	GeneSymbol	Ifit1	interferon-induced protein with tetratricopeptide repeats 1	ISG56|Ifi56	protein-coding	6.34	6.33	7.3	7.46	0.0058	6.335	7.38	1.045	2.06
10383192	Transcribed Locus	NA	NA	NA	Unknown	6.67	6.31	7.67	7.44	0.0380	6.49	7.555	1.065	2.09
10356262	GeneSymbol	Gm7609	predicted pseudogene 7609	EG665378	protein-coding	6.93	7.11	7.99	8.3	0.0245	7.02	8.145	1.125	2.18
10601385	GeneSymbol	Tlr13	toll-like receptor 13	AI666735|Gm713	protein-coding	7.01	6.76	7.81	8.16	0.0362	6.885	7.985	1.1	2.14
10574104	GeneSymbol	Nlrc5	NLR family, CARD domain containing 5	AK220210|NOD27	protein-coding	7.13	6.91	8.28	8	0.0244	7.02	8.14	1.12	2.17
10502791	GeneSymbol	Ifi44	interferon-induced protein 44	A430056A10Rik|AW261460|MTAP44|p44	protein-coding	5.46	5.72	6.46	6.47	0.0214	5.59	6.465	0.875	1.83
10527918	GeneSymbol	Mterf	mitochondrial transcription termination factor	4931431L11Rik|9230106K09Rik	protein-coding	6.4	6.14	7.38	7.12	0.0334	6.27	7.25	0.98	1.97
10541564	GeneSymbol	Clec4a3	C-type lectin domain family 4, member a3	3110037K17Rik|Dcir3|mDcir3	protein-coding	6.78	6.37	7.58	7.62	0.0381	6.575	7.6	1.025	2.03
10462390	GeneSymbol	Cd274	CD274 antigen	A530045L16Rik|B7h1|Pdcd1l1|Pdcd1lg1|Pdl1	protein-coding	6.78	6.83	8.05	7.67	0.0314	6.805	7.86	1.055	2.08
10574139	GeneSymbol	Nlrc5	NLR family, CARD domain containing 5	AK220210|NOD27	protein-coding	6.42	6.38	7.27	7.48	0.0118	6.4	7.375	0.975	1.97
10403871	GeneSymbol	Aoah	acyloxyacyl hydrolase	4930433E13Rik|MGC107583	protein-coding	6.37	6.19	7.15	7.31	0.0157	6.28	7.23	0.95	1.93
10574137	GeneSymbol	Nlrc5	NLR family, CARD domain containing 5	AK220210|NOD27	protein-coding	6.95	7.22	8.25	8.04	0.0251	7.085	8.145	1.06	2.08
10581813	GeneSymbol	Mlkl	mixed lineage kinase domain-like	9130019I15Rik	protein-coding	6.05	5.93	6.97	6.79	0.0145	5.99	6.88	0.89	1.85
10521667	GeneSymbol	Bst1	bone marrow stromal cell antigen 1	114/A10|A530073F09|BP-3|Bp3|Bsta1|CD157|Ly65	protein-coding	6.75	6.35	7.6	7.44	0.0459	6.55	7.52	0.97	1.96
10574098	GeneSymbol	Nlrc5	NLR family, CARD domain containing 5	AK220210|NOD27	protein-coding	7.52	7.52	8.58	8.65	0.0010	7.52	8.615	1.095	2.14
10344801	GeneSymbol	Cspp1	centrosome and spindle pole associated protein 1	2310020J12Rik|4930413O22Rik|AA387550|BC055915|Cspp2|Cspp3	protein-coding	7.1	6.7	7.86	7.92	0.0393	6.9	7.89	0.99	1.99
10519951	GeneSymbol	Pion	pigeon homolog (Drosophila)	A530088I07Rik	protein-coding	6.19	5.92	6.9	6.94	0.0240	6.055	6.92	0.865	1.82
10494271	GeneSymbol	Ctss	cathepsin S	-	protein-coding	8.83	8.84	10.23	9.96	0.0113	8.835	10.095	1.26	2.39
10603551	GeneSymbol	Cybb	cytochrome b-245, beta polypeptide	C88302|Cgd|Cyd|Nox2|gp91phox	protein-coding	8.98	8.77	9.93	10.34	0.0318	8.875	10.135	1.26	2.39
10574151	GeneSymbol	Nlrc5	NLR family, CARD domain containing 5	AK220210|NOD27	protein-coding	6.23	6.45	7.35	7.11	0.0319	6.34	7.23	0.89	1.85
10462618	GeneSymbol	Ifit3	interferon-induced protein with tetratricopeptide repeats 3	Ifi49|MGC107331	protein-coding	7.29	7.2	8.25	8.27	0.0021	7.245	8.26	1.015	2.02
10500335	GeneSymbol	Fcgr1	Fc receptor, IgG, high affinity I	AI323638|AV092959|CD64|FcgammaRI|IGGHAFC	protein-coding	6.59	6.49	7.4	7.5	0.0060	6.54	7.45	0.91	1.88
10382106	GeneSymbol	Gm885	predicted gene 885	Allergin-1|MGC179156|Mca32	protein-coding	6.37	6.29	7.22	7.2	0.0022	6.33	7.21	0.88	1.84
10474825	GeneSymbol	D2Ertd750e	DNA segment, Chr 2, ERATO Doi 750, expressed	1700025D04Rik|C15orf23|Traf4af1	protein-coding	7.32	7.17	8.39	8.11	0.0241	7.245	8.25	1.005	2.01
10379511	GeneSymbol	Ccl2	chemokine (C-C motif) ligand 2	AI323594|HC11|JE|MCAF|MCP-1|MCP1|SMC-CF|Scya2|Sigje	protein-coding	7.13	7.13	7.94	8.27	0.0275	7.13	8.105	0.975	1.97
10490150	GeneSymbol	Zbp1	Z-DNA binding protein 1	2010010H03Rik|Dai|Dlm1|mZaDLM	protein-coding	6.76	7	7.79	7.85	0.0169	6.88	7.82	0.94	1.92
10475517	GeneSymbol	AA467197	expressed sequence AA467197	MGC58382|Nmes1	protein-coding	5.77	5.97	6.66	6.68	0.0154	5.87	6.67	0.8	1.74
10439249	GeneSymbol	Parp14	poly (ADP-ribose) polymerase family, member 14	1600029O10Rik|BC021340|CoaSt6|KIAA1268|MGC29390|mKIAA1268	protein-coding	7.12	6.8	8	7.81	0.0367	6.96	7.905	0.945	1.93
10384458	GeneSymbol	Plek	pleckstrin	2010300B13Rik	protein-coding	7.48	7.24	8.45	8.26	0.0229	7.36	8.355	0.995	1.99
10483110	GeneSymbol	Ifih1	interferon induced with helicase C domain 1	9130009C22Rik|Helicard|Hlcd|MDA5|MGC90959	protein-coding	6.98	6.77	7.91	7.69	0.0260	6.875	7.8	0.925	1.90
10379535	GeneSymbol	Ccl8	chemokine (C-C motif) ligand 8	1810063B20Rik|AB023418|HC14|MCP-2|Mcp2|Scya8	protein-coding	6.98	6.78	7.88	7.71	0.0200	6.88	7.795	0.915	1.89
10466210	GeneSymbol	Ms4a6d	membrane-spanning 4-domains, subfamily A, member 6D	1110058E16Rik|AI447446|Ms4a11	protein-coding	7.5	7.31	8.36	8.4	0.0098	7.405	8.38	0.975	1.97
10541307	GeneSymbol	Usp18	ubiquitin specific peptidase 18	1110058H21Rik|AW047653|UBP43|Ubp15	protein-coding	6.91	7.09	7.91	7.93	0.0095	7	7.92	0.92	1.89
10385842	GeneSymbol	Rad50	RAD50 homolog (S. cerevisiae)	Mrell|Rad50l	protein-coding	7.87	7.62	8.9	8.6	0.0357	7.745	8.75	1.005	2.01
10534927	GeneSymbol	Pilra	paired immunoglobin-like type 2 receptor alpha	AV021745|FDF03	protein-coding	6.6	6.42	7.18	7.51	0.0471	6.51	7.345	0.835	1.78
10510167	GeneSymbol	Gm13051	predicted gene 13051	MGC117846|OTTMUSG00000010104|OTTMUSG00000010173	protein-coding	6.74	6.41	7.47	7.35	0.0415	6.575	7.41	0.835	1.78
10524621	GeneSymbol	Oasl2	2'-5' oligoadenylate synthetase-like 2	M1204|Mmu-OASL|Oasl	protein-coding	7.13	7.16	7.98	8.11	0.0054	7.145	8.045	0.9	1.87
10581069	GeneSymbol	Gm9853	predicted gene 9853	ENSMUSG00000051554	protein-coding	6.52	6.6	7.54	7.21	0.0408	6.56	7.375	0.815	1.76

10372410	GeneSymbol	Glipr1	GLI pathogenesis-related 1 (glioma)	2410114O14Rik|RTVP-1|RTVP1|mRTVP-1	protein-coding	6.59	6.38	7.28	7.27	0.0172	6.485	7.275	0.79	1.73
10547274	GeneSymbol	Zfp248	zinc finger protein 248	2810037F07Rik|E130106N01Rik|MGC100019	protein-coding	6.22	6.13	7.06	6.74	0.0487	6.175	6.9	0.725	1.65
10435457	GeneSymbol	Parp9	poly (ADP-ribose) polymerase family, member 9	AW214463|BC003281|Bagl|Bal|MGC7868	protein-coding	7.56	7.2	8.31	8.18	0.0456	7.38	8.245	0.865	1.82
10538832	GeneSymbol	Mad2l1	MAD2 mitotic arrest deficient-like 1 (yeast)	AA673185|MAD2|MGC113763	protein-coding	7.72	7.39	8.55	8.33	0.0467	7.555	8.44	0.885	1.85
10503170	GeneSymbol	Chd7	chromodomain helicase DNA binding protein 7	A730019I05Rik|Cycn|Cyn|Dz|Edy|Flo|Lda|Mt|Obt|Todo|WBE1|Whi	protein-coding	9	8.94	10.21	9.83	0.0320	8.97	10.02	1.05	2.07
10346191	GeneSymbol	Stat1	signal transducer and activator of transcription 1	2010005J02Rik|AA408197	protein-coding	7.85	7.69	8.81	8.54	0.0288	7.77	8.675	0.905	1.87
10423941	GeneSymbol	Ttc35	tetratricopeptide repeat domain 35	4921531G14Rik|AV060620|AW209495	protein-coding	10.29	9.89	11.41	11.11	0.0428	10.09	11.26	1.17	2.25
10530145	GeneSymbol	Tlr1	toll-like receptor 1	-	protein-coding	5.74	5.79	6.37	6.47	0.0072	5.765	6.42	0.655	1.57
10385526	GeneAccession	9930111J21Rik2	RIKEN cDNA 9930111J21 gene 2	9930111J21Rik|Irgb5|OTTMUSG00000005540|RP23-54N20.1	protein-coding	7.15	7.03	7.8	7.98	0.0178	7.09	7.89	0.8	1.74
10574163	GeneSymbol	Nlrc5	NLR family, CARD domain containing 5	AK220210|NOD27	protein-coding	6.38	6.47	7.24	7.05	0.0207	6.425	7.145	0.72	1.65
10351792	GeneSymbol	Slamf9	SLAM family member 9	2310026I04Rik|AI462096|CD2F-10|CD84-H1|Cd2f10|SF2001	protein-coding	7.19	7.01	7.88	7.89	0.0129	7.1	7.885	0.785	1.72
10531579	GeneSymbol	Hmgb1	high mobility group box 1	DEF|HMG-1|Hmg1|MGC103168|MGC103169|MGC117896|MGC117897|SBP-1|amphoterin|p30	protein-coding	9.86	9.54	10.92	10.61	0.0411	9.7	10.765	1.065	2.09
10607868	GeneSymbol	Tlr8	toll-like receptor 8	-	protein-coding	6.6	6.4	7.25	7.17	0.0222	6.5	7.21	0.71	1.64
10385513	GeneAccession	9930111J21Rik2	RIKEN cDNA 9930111J21 gene 2	9930111J21Rik|Irgb5|OTTMUSG00000005540|RP23-54N20.1	protein-coding	7.2	7.04	7.75	8.03	0.0412	7.12	7.89	0.77	1.71
10504470	GeneSymbol	Melk	maternal embryonic leucine zipper kinase	AI327312|MPK38|mKIAA0175	protein-coding	6.76	6.65	7.44	7.41	0.0062	6.705	7.425	0.72	1.65
10541555	GeneSymbol	Clec4a1	C-type lectin domain family 4, member a1	BC049354|Dcir4|MGC56880|mDcir4	protein-coding	7.23	6.93	7.8	7.87	0.0392	7.08	7.835	0.755	1.69
10458782	GeneSymbol	Pggt1b	protein geranylgeranyltransferase type I, beta subunit	2010207C17Rik|2610100E13|AI451237|AI551093|BGG1|GGT1	protein-coding	6.94	7.06	7.69	7.8	0.0117	7	7.745	0.745	1.68
10420426	GeneSymbol	F630043A04Rik	RIKEN cDNA F630043A04 gene	Ska3	protein-coding	6.47	6.36	7.24	6.95	0.0483	6.415	7.095	0.68	1.60
10606376	GeneSymbol	2610002M06Rik	RIKEN cDNA 2610002M06 gene	8430408J07Rik|AI876413|Chmp1b2	protein-coding	6.43	6.19	7.07	6.88	0.0491	6.31	6.975	0.665	1.59
10473125	GeneSymbol	Itga4	integrin alpha 4	CD49D	protein-coding	7.46	7.45	8.15	8.33	0.0129	7.455	8.24	0.785	1.72
10458938	GeneSymbol	Gm8594	predicted gene 8594	EG667360	pseudo	6.76	6.6	7.31	7.45	0.0223	6.68	7.38	0.7	1.62
10591881	GeneSymbol	Hmgb1	high mobility group box 1	DEF|HMG-1|Hmg1|MGC103168|MGC103169|MGC117896|MGC117897|SBP-1|amphoterin|p30	protein-coding	10.17	9.99	11.29	10.98	0.0277	10.08	11.135	1.055	2.08
10383212	Transcribed Locus	NA	NA	NA	Unknown	7.75	7.6	8.41	8.51	0.0129	7.675	8.46	0.785	1.72
10383214	GeneSymbol	Rnf213	ring finger protein 213	6030403J01|D11Ertd759e|KIAA1554|mKIAA1554	protein-coding	7.52	7.36	8.09	8.31	0.0306	7.44	8.2	0.76	1.69
10344895	GeneSymbol	Gm7560	predicted gene 7560	EG665262	pseudo	6.41	6.15	6.9	6.94	0.0397	6.28	6.92	0.64	1.56
10512067	GeneSymbol	Ddx58	DEAD (Asp-Glu-Ala-Asp) box polypeptide 58	6430573D20Rik|C330021E21|RIG-I	protein-coding	7.75	7.51	8.39	8.39	0.0240	7.63	8.39	0.76	1.69
10368222	GeneSymbol	4930444G20Rik	RIKEN cDNA 4930444G20 gene	-	protein-coding	6.43	6.34	6.97	7.06	0.0101	6.385	7.015	0.63	1.55
10389627	GeneSymbol	Rad51c	RAD51 homolog c (S. cerevisiae)	Rad51l2	protein-coding	6.19	6.08	6.86	6.62	0.0445	6.135	6.74	0.605	1.52
10374842	GeneSymbol	Ccdc88a	coiled coil domain containing 88A	A430106J12Rik|AI848406|Ape|C130096N06Rik|C330012F17Rik|D130005J21Rik|Girdin|Giv|Hkrp1	protein-coding	6.38	6.18	6.91	6.88	0.0260	6.28	6.895	0.615	1.53
10502029	GeneSymbol	Larp7	La ribonucleoprotein domain family, member 7	C330027G06Rik|D3Wsu161e	protein-coding	7.88	7.69	8.64	8.45	0.0299	7.785	8.545	0.76	1.69
10439268	GeneSymbol	Dtx3l	deltex 3-like (Drosophila)	AU042200|BC023741|MGC103262	protein-coding	7.35	7.1	7.9	7.96	0.0317	7.225	7.93	0.705	1.63
10594774	GeneSymbol	Ccnb2	cyclin B2	CycB2	protein-coding	8.99	8.86	9.87	9.71	0.0139	8.925	9.79	0.865	1.82
10443980	GeneSymbol	Myo1f	myosin IF	C330006B10Rik	protein-coding	6.95	7.05	7.56	7.79	0.0328	7	7.675	0.675	1.60
10397543	GeneAccession	Gm5662	predicted gene 5662	EG435337	protein-coding	7.6	7.28	8.16	8.15	0.0466	7.44	8.155	0.715	1.64
10424221	GeneSymbol	Wdr67	WD repeat domain 67	4B3|D330013L20Rik|Gm85|MGC76603	protein-coding	7.36	7.21	7.97	8	0.0117	7.285	7.985	0.7	1.62
10401829	GeneAccession	Gm5662	predicted gene 5662	EG435337	protein-coding	7.38	7.1	7.93	7.94	0.0383	7.24	7.935	0.695	1.62
10487208	GeneSymbol	Atp8b4	ATPase, class I, type 8B, member 4	A530043E15|Im|MGC161341	protein-coding	5.91	6.11	6.5	6.67	0.0484	6.01	6.585	0.575	1.49
10547088	GeneSymbol	Mbd4	methyl-CpG binding domain protein 4	Med1	protein-coding	6.33	6.53	7.1	6.99	0.0328	6.43	7.045	0.615	1.53
10574143	GeneSymbol	Nlrc5	NLR family, CARD domain containing 5	AK220210|NOD27	protein-coding	6.08	6.14	6.68	6.7	0.0030	6.11	6.69	0.58	1.49
10492888	Transcribed Locus	NA	NA	NA	Unknown	7.64	7.43	8.34	8.16	0.0354	7.535	8.25	0.715	1.64
10376060	GeneSymbol	Irf1	interferon regulatory factor 1	AU020929|Irf-1	protein-coding	6.85	6.8	7.47	7.47	0.0015	6.825	7.47	0.645	1.56
10503107	GeneSymbol	6330407A03Rik	RIKEN cDNA 6330407A03 gene	-	miscRNA	5.98	6.12	6.58	6.65	0.0187	6.05	6.615	0.565	1.48
10392808	GeneSymbol	Cd300ld	CD300 molecule-like family member d	4732429D16Rik|CLM5|LMIR4|MAIR-IV|clm-5	protein-coding	6.47	6.53	7.15	7.06	0.0079	6.5	7.105	0.605	1.52
10353475	GeneSymbol	Eif3m	eukaryotic translation initiation factor 3, subunit M	Ga17|MGC118449|Pcid1	protein-coding	7.87	7.93	8.75	8.5	0.0300	7.9	8.625	0.725	1.65
10372648	GeneSymbol	Lyz2	lysozyme 2	AI326280|Lys|Lyzs|Lzm|Lzm-s1|Lzp	protein-coding	11.15	10.74	11.94	11.95	0.0396	10.945	11.945	1	2.00
10358224	GeneSymbol	Ptprc	protein tyrosine phosphatase, receptor type, C	B220|CD45R|Cd45|Ly-5|Lyt-4|T200|loc	protein-coding	7.94	7.75	8.46	8.65	0.0340	7.845	8.555	0.71	1.64
10477649	GeneSymbol	Acss2	acyl-CoA synthetase short-chain family member 2	1110017C11Rik|ACAS|Acas1|Acas2|AceCS1|Acs1	protein-coding	7.84	7.58	8.45	8.35	0.0384	7.71	8.4	0.69	1.61
10462613	GeneSymbol	Ifit2	interferon-induced protein with tetratricopeptide repeats 2	AV302338|Ifi54	protein-coding	6.49	6.26	6.91	6.98	0.0417	6.375	6.945	0.57	1.48
10496771	GeneSymbol	Mcoln2	mucolipin 2	3300002C04Rik|AI549968|C86638|TRPML2	protein-coding	6.23	6.39	6.77	6.96	0.0466	6.31	6.865	0.555	1.47
10382104	Transcribed Locus	NA	NA	NA	Unknown	10.96	11.26	12.21	11.95	0.0394	11.11	12.08	0.97	1.96
10363265	GeneSymbol	Lims1	LIM and senescent cell antigen-like domains 1	2310016J22Rik|4921524A02Rik|AI507642|AU021743|AW551584|C430041B13Rik|Lims1l|PINCH|PINCH1	protein-coding	8.61	8.36	9.33	9.12	0.0454	8.485	9.225	0.74	1.67
10569890	GeneSymbol	Aida	axin interactor, dorsalization associated	2610208M17Rik|BC004835|MGC101949|MGC6869	protein-coding	8.84	8.53	9.49	9.39	0.0435	8.685	9.44	0.755	1.69
10477250	GeneSymbol	Hck	hemopoietic cell kinase	AI849071|Bmk|MGC18625	protein-coding	7	6.92	7.58	7.55	0.0049	6.96	7.565	0.605	1.52
10475648	GeneSymbol	Dtwd1	DTW domain containing 1	1810033A06Rik	protein-coding	7.43	7.29	8.04	7.95	0.0167	7.36	7.995	0.635	1.55
10542738	GeneSymbol	Rassf8	Ras association (RalGDS/AF-6) domain family (N-terminal) member 8	5133400D11Rik|AA815821|AW123240|Hoj1|MGC144731|MGC144732|mHoj-1	protein-coding	8.16	7.87	8.69	8.72	0.0419	8.015	8.705	0.69	1.61
10581434	GeneSymbol	Dpep2	dipeptidase 2	F630103D06Rik|MBD-2	protein-coding	7.98	7.87	8.5	8.7	0.0274	7.925	8.6	0.675	1.60
10358589	GeneSymbol	Hmcn1	hemicentin 1	EG545370|Gm201	protein-coding	7.65	7.5	8.24	8.2	0.0142	7.575	8.22	0.645	1.56
10505630	GeneSymbol	Snapc3	small nuclear RNA activating complex, polypeptide 3	1810020H02Rik|4930558A07Rik|5031401C21Rik|AI414457|E030018J20Rik	protein-coding	9.61	9.42	10.29	10.35	0.0150	9.515	10.32	0.805	1.75
10344799	GeneSymbol	Cspp1	centrosome and spindle pole associated protein 1	2310020J12Rik|4930413O22Rik|AA387550|BC055915|Cspp2|Cspp3	protein-coding	7.82	7.8	8.45	8.49	0.0011	7.81	8.47	0.66	1.58
10376144	GeneSymbol	Fnip1	folliculin interacting protein 1	A730024A03Rik|AI838773|AW557298|KIAA1961|MGC100017	protein-coding	8.39	8.2	8.91	9.07	0.0305	8.295	8.99	0.695	1.62
10383233	GeneSymbol	Rnf213	ring finger protein 213	6030403J01|D11Ertd759e|KIAA1554|mKIAA1554	protein-coding	8.06	7.95	8.54	8.8	0.0422	8.005	8.67	0.665	1.59
10427918	GeneSymbol	Fam105a	family with sequence similarity 105, member A	9830126M18|MGC59004	protein-coding	6.76	6.54	7.2	7.2	0.0377	6.65	7.2	0.55	1.46
10401841	GeneSymbol	Dio2	deiodinase, iodothyronine, type II	5DII|AI324267|DIOII	protein-coding	6.88	6.86	7.36	7.51	0.0175	6.87	7.435	0.565	1.48
10445119	GeneSymbol	H2-M3	histocompatibility 2, M region locus 3	H-2M3|Hmt|M3a|R4B2	protein-coding	6.77	6.85	7.42	7.32	0.0128	6.81	7.37	0.56	1.47
10502240	GeneSymbol	Npnt	nephronectin	1110009H02Rik|AA682063|AI314031|Nctn|POEM	protein-coding	8.09	8	8.59	8.79	0.0277	8.045	8.69	0.645	1.56
10413059	GeneSymbol	Vcl	vinculin	9430097D22|AA571387|AI462105|AW545629	protein-coding	8.78	8.51	9.4	9.27	0.0441	8.645	9.335	0.69	1.61
10446282	GeneSymbol	Emr1	EGF-like module containing, mucin-like, hormone receptor-like sequence 1	DD7A5-7|EGF-TM7|F4/80|Gpf480|Ly71|TM7LN3	protein-coding	8.16	8.02	8.68	8.78	0.0176	8.09	8.73	0.64	1.56
10410560	GeneSymbol	Trip13	thyroid hormone receptor interactor 13	2410002G23Rik|D13Ertd328e	protein-coding	6.83	6.87	7.51	7.27	0.0472	6.85	7.39	0.54	1.45
10544732	GeneSymbol	Skap2	src family associated phosphoprotein 2	2610021A10Rik|AA960083|BB137539|RA70|SKAP-HOM|Saps|Scap2|mSKAP55R	protein-coding	7.29	7.18	7.8	7.81	0.0093	7.235	7.805	0.57	1.48
10397085	GeneSymbol	Rbm25	RNA binding motif protein 25	2600011C06Rik|2610015J01Rik|A130095G20Rik|AI159652|AL023075|AU043498|RNPC7	protein-coding	9.49	9.2	10.09	10.07	0.0370	9.345	10.08	0.735	1.66
10444258	GeneSymbol	Psmb8	proteasome (prosome, macropain) subunit, beta type 8 (large multifunctional peptidase 7)	Lmp-7|Lmp7	protein-coding	6.94	7.05	7.47	7.62	0.0274	6.995	7.545	0.55	1.46
10574102	GeneSymbol	Nlrc5	NLR family, CARD domain containing 5	AK220210|NOD27	protein-coding	7.84	7.7	8.47	8.29	0.0332	7.77	8.38	0.61	1.53
10418004	GeneSymbol	Ap3m1	adaptor-related protein complex 3, mu 1 subunit	1200013D09Rik|C78982|R75378	protein-coding	8.34	8.33	8.99	8.98	0.0001	8.335	8.985	0.65	1.57
10565589	GeneSymbol	Hmgb1	high mobility group box 1	DEF|HMG-1|Hmg1|MGC103168|MGC103169|MGC117896|MGC117897|SBP-1|amphoterin|p30	protein-coding	10.43	10.15	11.18	11	0.0407	10.29	11.09	0.8	1.74
10383206	Transcribed Locus	NA	NA	NA	Unknown	7.31	7.27	7.75	7.96	0.0340	7.29	7.855	0.565	1.48
10478678	GeneSymbol	Cd40	CD40 antigen	AI326936|Bp50|GP39|HIGM1|IGM|IMD3|T-BAM|TRAP|Tnfrsf5|p50	protein-coding	7.19	7.39	7.84	7.87	0.0306	7.29	7.855	0.565	1.48
10444780	GeneSymbol	H2-D1	histocompatibility 2, D region locus 1	H-2D|H2-D	protein-coding	10.39	10.3	11.17	11.12	0.0041	10.345	11.145	0.8	1.74
10507273	GeneSymbol	Pik3r3	phosphatidylinositol 3 kinase, regulatory subunit, polypeptide 3 (p55)	AA414954|p55pik	protein-coding	6.98	6.86	7.39	7.52	0.0263	6.92	7.455	0.535	1.45
10580582	GeneSymbol	4933436C20Rik	RIKEN cDNA 4933436C20 gene	-	miscRNA	7.71	7.46	8.16	8.18	0.0430	7.585	8.17	0.585	1.50
10501661	GeneAccession	Srsf3	serine/arginine-rich splicing factor 3	AL024116|Sfrs3|X16	protein-coding	10.06	9.9	10.85	10.64	0.0285	9.98	10.745	0.765	1.70
10494662	GeneSymbol	Ywhah	tyrosine 3-monooxygenase/tryptophan 5-monooxygenase activation protein, eta polypeptide	-	protein-coding	10.06	9.85	10.73	10.7	0.0189	9.955	10.715	0.76	1.69
10539773	GeneSymbol	Gfpt1	glutamine fructose-6-phosphate transaminase 1	2810423A18Rik|AI324119|AI449986|GFA|GFAT|GFAT1|GFAT1m|Gfpt	protein-coding	7.2	7.07	7.72	7.63	0.0208	7.135	7.675	0.54	1.45

<table>
<tr><td>10584334</td><td>GeneSymbol</td><td>Siae</td><td>sialic acid acetylesterase</td><td>LSE|Ysg2</td><td>protein-coding</td><td>7.18</td><td>7</td><td>7.61</td><td>7.64</td><td>0.0279</td><td>7.09</td><td>7.625</td><td>0.535</td><td>1.45</td></tr>
<tr><td>10510254</td><td>GeneSymbol</td><td>Fv1</td><td>Friend virus susceptibility 1</td><td>Fv-1|Rv-1|Rv1</td><td>protein-coding</td><td>7.16</td><td>7.17</td><td>7.82</td><td>7.59</td><td>0.0426</td><td>7.165</td><td>7.705</td><td>0.54</td><td>1.45</td></tr>
<tr><td>10446771</td><td>GeneSymbol</td><td>Lclat1</td><td>lysocardiolipin acyltransferase 1</td><td>AI181996|Agpat8|Alcat1|Gm91|Lycat</td><td>protein-coding</td><td>8.95</td><td>8.71</td><td>9.4</td><td>9.58</td><td>0.0480</td><td>8.83</td><td>9.49</td><td>0.66</td><td>1.58</td></tr>
<tr><td>10421768</td><td>GeneSymbol</td><td>Akap11</td><td>A kinase (PRKA) anchor protein 11</td><td>6330501D17Rik|AKAP220|Gm80|PRKA11|mKIAA0629</td><td>protein-coding</td><td>8.71</td><td>8.53</td><td>9.28</td><td>9.24</td><td>0.0201</td><td>8.62</td><td>9.26</td><td>0.64</td><td>1.56</td></tr>
<tr><td>10543959</td><td>GeneSymbol</td><td>Ptn</td><td>pleiotrophin</td><td>HARP|HB-GAM|HBBN|HBGF-8|HBNF|OSF|Osf-1|Osf1</td><td>protein-coding</td><td>7.58</td><td>7.59</td><td>8.12</td><td>8.17</td><td>0.0021</td><td>7.585</td><td>8.145</td><td>0.56</td><td>1.47</td></tr>
<tr><td>10551760</td><td>GeneSymbol</td><td>Zfp84</td><td>zinc finger protein 84</td><td>2210410P13Rik|4633401C23Rik|AI415447|BB081605|C86188|KRAB18|Zfp69</td><td>protein-coding</td><td>7.77</td><td>7.87</td><td>8.47</td><td>8.32</td><td>0.0237</td><td>7.82</td><td>8.395</td><td>0.575</td><td>1.49</td></tr>
<tr><td>10405731</td><td>GeneSymbol</td><td>4933434E20Rik</td><td>RIKEN cDNA 4933434E20 gene</td><td>5730552F22Rik|AI462154|NICE-3|NS5ATP4</td><td>protein-coding</td><td>8.58</td><td>8.58</td><td>9.34</td><td>9.08</td><td>0.0400</td><td>8.58</td><td>9.21</td><td>0.63</td><td>1.55</td></tr>
<tr><td>10407766</td><td>GeneSymbol</td><td>Lgals8</td><td>lectin, galactose binding, soluble 8</td><td>1200015E08Rik|AI326142|D13Ertd524e|Lgals-8</td><td>protein-coding</td><td>8.38</td><td>8.26</td><td>9.01</td><td>8.85</td><td>0.0258</td><td>8.32</td><td>8.93</td><td>0.61</td><td>1.53</td></tr>
<tr><td>10371277</td><td>GeneSymbol</td><td>B230315N10Rik</td><td>RIKEN cDNA B230315N10 gene</td><td>MGC19022</td><td>protein-coding</td><td>8.06</td><td>7.88</td><td>8.47</td><td>8.63</td><td>0.0405</td><td>7.97</td><td>8.55</td><td>0.58</td><td>1.49</td></tr>
<tr><td>10383152</td><td>Transcribed Locus</td><td>NA</td><td>NA</td><td>NA</td><td>Unknown</td><td>7.63</td><td>7.65</td><td>8.1</td><td>8.29</td><td>0.0284</td><td>7.64</td><td>8.195</td><td>0.555</td><td>1.47</td></tr>
<tr><td>10462454</td><td>GeneSymbol</td><td>Uhrf2</td><td>ubiquitin-like, containing PHD and RING finger domains 2</td><td>2310065A22Rik|AI426270|AW214556|D130071B19Rik|Nirf</td><td>protein-coding</td><td>7.94</td><td>7.8</td><td>8.53</td><td>8.33</td><td>0.0444</td><td>7.87</td><td>8.43</td><td>0.56</td><td>1.47</td></tr>
<tr><td>10445781</td><td>GeneSymbol</td><td>Trem2</td><td>triggering receptor expressed on myeloid cells 2</td><td>Trem2a|Trem2b|Trem2c</td><td>protein-coding</td><td>7.58</td><td>7.75</td><td>8.12</td><td>8.3</td><td>0.0479</td><td>7.665</td><td>8.21</td><td>0.545</td><td>1.46</td></tr>
<tr><td>10522596</td><td>GeneSymbol</td><td>Tmem165</td><td>transmembrane protein 165</td><td>AV026557|Tpardl|Tparl|pFT27</td><td>protein-coding</td><td>9.86</td><td>9.62</td><td>10.48</td><td>10.31</td><td>0.0469</td><td>9.74</td><td>10.395</td><td>0.655</td><td>1.57</td></tr>
<tr><td>10451650</td><td>GeneSymbol</td><td>Nfya</td><td>nuclear transcription factor-Y alpha</td><td>AA407810|Cbf-b|SEZ-10|SEZ10</td><td>protein-coding</td><td>8.35</td><td>8.23</td><td>8.95</td><td>8.73</td><td>0.0482</td><td>8.29</td><td>8.84</td><td>0.55</td><td>1.46</td></tr>
<tr><td>10485395</td><td>GeneSymbol</td><td>Trim44</td><td>tripartite motif-containing 44</td><td>Dipb|MGC102054|Mc7</td><td>protein-coding</td><td>8.27</td><td>8.26</td><td>8.91</td><td>8.71</td><td>0.0321</td><td>8.265</td><td>8.81</td><td>0.545</td><td>1.46</td></tr>
<tr><td>10348004</td><td>GeneSymbol</td><td>Psmd1</td><td>proteasome (prosome, macropain) 26S subunit, non-ATPase, 1</td><td>2410026J11Rik|P112|S1</td><td>protein-coding</td><td>10.42</td><td>10.14</td><td>11</td><td>10.91</td><td>0.0443</td><td>10.28</td><td>10.955</td><td>0.675</td><td>1.60</td></tr>
<tr><td>10502058</td><td>GeneAccession</td><td>Ap1ar</td><td>adaptor-related protein complex 1 associated regulatory protein</td><td>AA407621|C78909|MGC7434</td><td>protein-coding</td><td>9.75</td><td>9.65</td><td>10.43</td><td>10.23</td><td>0.0301</td><td>9.7</td><td>10.33</td><td>0.63</td><td>1.55</td></tr>
<tr><td>10444830</td><td>GeneSymbol</td><td>H2-Q7</td><td>histocompatibility 2, Q region locus 7</td><td>H-2Q7|Ped|Q9|Qa-2|Qa-7|Qa7</td><td>protein-coding</td><td>10.56</td><td>10.46</td><td>11.14</td><td>11.23</td><td>0.0098</td><td>10.51</td><td>11.185</td><td>0.675</td><td>1.60</td></tr>
<tr><td>10574436</td><td>GeneSymbol</td><td>Khdrbs1</td><td>KH domain containing, RNA binding, signal transduction associated 1</td><td>Sam68|p62</td><td>protein-coding</td><td>9.14</td><td>8.93</td><td>9.58</td><td>9.64</td><td>0.0342</td><td>9.035</td><td>9.61</td><td>0.575</td><td>1.49</td></tr>
<tr><td>10366004</td><td>GeneSymbol</td><td>Atp2b1</td><td>ATPase, Ca++ transporting, plasma membrane 1</td><td>2810442I22Rik|E130111D10Rik|Pmca1</td><td>protein-coding</td><td>9.82</td><td>9.62</td><td>10.31</td><td>10.34</td><td>0.0268</td><td>9.72</td><td>10.325</td><td>0.605</td><td>1.52</td></tr>
<tr><td>10586168</td><td>GeneSymbol</td><td>Snord16a</td><td>small nucleolar RNA, C/D box 16A</td><td>MBII-98</td><td>snoRNA</td><td>9.05</td><td>8.92</td><td>9.5</td><td>9.55</td><td>0.0162</td><td>8.985</td><td>9.525</td><td>0.54</td><td>1.45</td></tr>
<tr><td>10364280</td><td>GeneSymbol</td><td>Pttg1ip</td><td>pituitary tumor-transforming 1 interacting protein</td><td>1810010L20Rik|AI314311|AU018448|C79540|MGC36923</td><td>protein-coding</td><td>9.57</td><td>9.42</td><td>10.07</td><td>10.05</td><td>0.0175</td><td>9.495</td><td>10.06</td><td>0.565</td><td>1.48</td></tr>
<tr><td>10413695</td><td>GeneSymbol</td><td>Pbrm1</td><td>polybromo 1</td><td>2610016F04Rik|AI507524|BAF180|Pb1</td><td>protein-coding</td><td>9.96</td><td>9.76</td><td>10.48</td><td>10.41</td><td>0.0313</td><td>9.86</td><td>10.445</td><td>0.585</td><td>1.50</td></tr>
<tr><td>10493292</td><td>GeneSymbol</td><td>2810403A07Rik</td><td>RIKEN cDNA 2810403A07 gene</td><td>A430106P18Rik|AI256352|AI451678|Blom7|Kiaa0907</td><td>protein-coding</td><td>9.35</td><td>9.11</td><td>9.78</td><td>9.76</td><td>0.0463</td><td>9.23</td><td>9.77</td><td>0.54</td><td>1.45</td></tr>
<tr><td>10363703</td><td>GeneSymbol</td><td>Jmjd1c</td><td>jumonji domain containing 1C</td><td>5430433L24Rik|D630035I23Rik|Jmjdic|TRIP8</td><td>protein-coding</td><td>9.19</td><td>8.99</td><td>9.65</td><td>9.59</td><td>0.0367</td><td>9.09</td><td>9.62</td><td>0.53</td><td>1.44</td></tr>
<tr><td>10471586</td><td>GeneSymbol</td><td>Hspa5</td><td>heat shock protein 5</td><td>AL022860|AU019543|Bip|D2Wsu141e|D2Wsu17e|Grp78|Hsce70|SEZ-7|Sez7|mBiP</td><td>protein-coding</td><td>10.36</td><td>10.23</td><td>10.94</td><td>10.84</td><td>0.0185</td><td>10.295</td><td>10.89</td><td>0.595</td><td>1.51</td></tr>
<tr><td>10572747</td><td>GeneSymbol</td><td>Tpm4</td><td>tropomyosin 4</td><td>2610528G24Rik</td><td>protein-coding</td><td>10.73</td><td>10.48</td><td>11.18</td><td>11.13</td><td>0.0497</td><td>10.605</td><td>11.155</td><td>0.55</td><td>1.46</td></tr>
<tr><td>10517508</td><td>GeneSymbol</td><td>C1qb</td><td>complement component 1, q subcomponent, beta polypeptide</td><td>-</td><td>protein-coding</td><td>10.44</td><td>10.37</td><td>10.84</td><td>11.04</td><td>0.0371</td><td>10.405</td><td>10.94</td><td>0.535</td><td>1.45</td></tr>
<tr><td>10385599</td><td>GeneSymbol</td><td>Canx</td><td>calnexin</td><td>1110069N15Rik|AI988026|Cnx|D11Ertd153e</td><td>protein-coding</td><td>11.3</td><td>11.16</td><td>11.77</td><td>11.83</td><td>0.0174</td><td>11.23</td><td>11.8</td><td>0.57</td><td>1.48</td></tr>
</table>

Table S4. Table showing the full data set for upstream regulators shown in Table 1.

Upstream Regulator	Molecule Type	Predicted Activation State	Activation z-score	p-value of overlap	Target molecules in dataset
**Type I IFN responses**					
Ifnar	group	Activated	4.5	1.4E-27	Ccl2,CD274,CD40,CXCL10,CXCL9,DDX58,HLA-B,HLAG,IFIH1,IFIT1B,IFIT2,IFIT3,IRF1,IRGM,ITGA4,NLRC5,PSMB8,Rnf213,STAT1,USP18,ZBP1
IFNB1	cytokine	Activated	3.8	1.4E-23	Ccl2,CCR5,CD274,CD40,CXCL10,CXCL9,DDX58,GBP4,Gbp6 (includes others),GBP7,Gbp8,Gvin1 (includes others),IFIH1,IFIT1B,IFIT2,IFIT3,IRF1,Irg1,IRGM,STAT1,Tgtp1/Tgtp2,Tlr13,Trim30a/Trim30d,USP18,VCL,ZBP1
IRF7	transcription regulator	Activated	4.3	5.9E-20	CD40,CXCL10,CXCL9,DDX58,FCGR1A,GBP4,IFI44,IFIH1,IFIT1B,IFIT2,IFIT3,IRF1,IRGM,Oasl2,PARP14,PSMB8,STAT1,Trim30a/Trim30d,USP18,ZBP1
STAT1	transcription regulator	Activated	4.3	2.4E-17	Ccl2,Ccl8,CD274,CD40,CXCL10,CXCL9,FCGR1A,Gbp6 (includes others),HERC6,IFIT1B,IFIT2,IFIT3,IRF1,Irg1,IRGM,PSMB8,PTN,Rnf213,STAT1,Tgtp1/Tgtp2,USP18
IRF3	transcription regulator	Activated	3.6	7.4E-16	Ccl2,CXCL10,CXCL9,DDX58,FCGR1A,IFI44,IFIH1,IFIT1B,IFIT2,IFIT3,IRGM,Oasl2,PARP14,STAT1,Trim30a/Trim30d,USP18,ZBP1
TLR3	transmembrane receptor	Activated	3.6	2.4E-14	Ccl2,CD274,CD40,CXCL10,CXCL9,DDX58,GBP4,IFI44,IFIH1,IFIT2,IFIT3,Iigp1,Irg1,Oasl2,PIK3R3,RGS1,STAT1,USP18
Interferon alpha	group	Activated	4.4	5.1E-14	CCR5,CD40,CXCL10,CXCL9,DDX58,FCGR1A,HLA-B,HLA-G,FIH1,IFIT1B,IFIT2,IFIT3,IRF1,ITGA4,PSMB8,STAT1,Tgtp1/Tgtp2,TLR1,USP18,ZBP1
IFNL1	cytokine	Activated	3.4	7.5E-14	CD40,CXCL10,CXCL9,DDX58,DDX60,HERC6,IFI44,IFIH1,IFIT2,IFIT3,STAT1,USP18
IFNAR1	transmembrane receptor	Activated	2.8	3.6E-13	CD40,CXCL10,CXCL9,HLA-B,IFI44,IFIH1,IFIT1B,IFIT2,IFIT3,Irg1,Oasl2,STAT1,USP18
IFN Beta	group	Activated	3.4	6.5E-13	CD274,CD40,CXCL10,DDX58,HLA-B,HLA-G,IFIH1,IFIT1B,IFIT2,IRF1,STAT1,USP18,ZBP1
IFNA2	cytokine	Activated	3.7	2.0E-12	CXCL10,CXCL9,DDX58,DDX60,EMR1,HERC6,HLA-B,IFI44,IFIH1,IFIT2,IFIT3,IRF1,PARP9,STAT1,USP18,ZBP1
IFN alpha/beta	group	Activated	3.3	5.6E-12	CCR2,CD40,CXCL10,CXCL9,IFIT2,IFIT3,IRF1,IRGM,STAT1,Tgtp1/Tgtp2,TLR1
IFNA1/IFNA13	cytokine	Activated	2.9	8.5E-12	CD40,CXCL10,CXCL9,IFIH1,IFIT2,IRF1,STAT1,Tgtp1/Tgtp2,USP18, ZBP1
TLR4	transmembrane receptor	Activated	3.3	1.3E-11	Ccl2,CCR5,CD274,CD40,CXCL10,CXCL9,HMGB1,IFIT1B,IFIT2,IFIT3,Iigp1,IRF1,IRGM,PLEK,RGS1,STAT1,TREM2
IFN type 1	group	Activated	2.9	2.2E-11	Ccl2,CXCL10,CXCL9,DDX58,IFIH1,IFIT1B,IFIT2,IRF1,STAT1
DDX58 (RIG-I)	enzyme	Activated	2.0	4.0E-11	CXCL10,DDX58,IFI44,IFIH1,IFIT1B,IFIT2,IFIT3,IRF1,STAT1
IFNE	cytokine	Activated	2.4	8.3E-11	CD40,CXCL10,CXCL9,IFIH1,IFIT2,STAT1,USP18,ZBP1
IFNK	cytokine	Activated	2.2	1.1E-10	CD40,CXCL10,CXCL9,IFIH1,IRF1,STAT1,ZBP1
IKBKE	kinase		0.1	9.7E-10	Ccl2,CXCL10,HLA-B,IFIH1,IFIT2,IFIT3,Irg1,STAT1
**Negative regulators of Type 1 IFN responses**					
SOCS1	other	Inhibited	-3.9	4.6E-20	Ccl2,CD40,CXCL10,CXCL9,DDX58,Gbp6 (includes others),GBP7,Gvin1 (includes others),IFI44,IFIH1,IFIT1B,IFIT2,IFIT3,Iigp1,IRF1,Irg1,Oasl2,STAT1
TRIM24	transcription regulator	Inhibited	-4.1	9.4E-20	CXCL10,DDX58,DDX60,GBP4,HERC6,IFI44,IFIH1,IFIT1B,IFIT2,IFIT3,Iigp1,IRF1,IRGM,PSMB8,STAT1,Tgtp1/Tgtp2,USP18
mir-21	microRNA	Inhibited	-3.8	1.0E-17	AOAH,CD180,CD274,CXCL10,CXCL9,FCGR1A,Gbp6 (includes others),GBP7,Gbp8,Gvin1 (includes others),Iigp1,IRF1,IRGM,NLRC5,STAT1,Tgtp1/Tgtp2,TLR1,TREM2
**Cytokines**					
IFNG	cytokine	Activated	6.3	1.1E-21	BST1,C1QB,Ccl2,Ccl8,CCR2,CCR5,CD274,CD40,CTSS,CXCL10,CXCL9,CYBB,DDX58,DTX3L,FCGR1A,FCGR3A,GBP4,Gbp6 (includes others),GBP7,Gbp8,Gvin1 (includes others),HCK,HERC6,HLA-B,HLA-G,HMGB1,HSPD1,IFI44,IFIH1,IFIT2,IFIT3,Iigp1,IRF1,Irg1,IRGM,NLRC5,PARP9,PLEK,PSMB8,PTN,STAT1,Tgtp1/Tgtp2,TLR1,TREM2,USP18
IL12 (complex)	complex		1.8	5.7E-11	Ccl2,CCR2,CCR5,CXCL10,CXCL9,EMR1,IFIH1,IFIT1B,IFIT2,Iigp1,IRF1,ITGA4,Tgtp1/Tgtp2,Trim30a/Trim30d
IL6	cytokine	Activated	3.9	6.7E-10	Ccl2,CCNB2,CCR2,CCR5,CD40,CXCL10,CXCL9,CYBB,EMR1,FCGR1A,HLA-B,HSPA5,IFIT1B,IFIT2,IRF1,LY86,Lyz1/Lyz2,MAD2L1,PSMB8,PTPRC,STAT1,TLR1
**T cell responses**					
DOCK8	other	Activated	3.2	3.6E-11	CD40,CXCL10,CXCL9,IFIT1B,IFIT2,IFIT3,Iigp1,IRF1,RGS1,STAT1
TCR	complex		0.5	5.3E-11	CCR2,CCR5,CXCL10,CXCL9,DDX58,GBP4,HSPA5,HSPD1,IFI44,IFIH1,IFIT2,IFIT3,IRF1,PIK3R3,PTPRC,STAT1
**Apoptosis**					
PARP1	enzyme	Activated	3.1	1.6E-10	Ccl2,CD274,CXCL10,CXCL9,GBP4,Gbp6 (includes others),GBP7,Iigp1,STAT1,Tgtp1/Tgtp2
**M2**					
STAT3	transcription regulator	Activated	2.3	2.5E-10	Ccl8,CCR5,CD274,CD40,CXCL10,CXCL9,FCGR1A,GLIPR1,HLA-B,IFI44,IFIH1,IFIT1B,IFIT3,IRF1,Lyz1/Lyz2,PSMB8,PTN,STAT1
**Autophagy**					
IRGM	other	Inhibited	-2.8	3.8E-10	CCNB2,CXCL10,CXCL9,IFIT1B,IFIT2,IFIT3,Iigp1,USP18
**Other**					
PLK4	kinase	Activated	2.6	2.2E-10	CD40,CXCL10,CXCL9,IFIT2,Iigp1,IRF1,RGS1
PLK2	kinase	Activated	2.6	3.0E-10	CD40,CXCL10,CXCL9,IFIT2,Iigp1,IRF1,RGS1
FZD9	G-protein coupled receptor	Activated	2.4	9.2E-10	Ccl2,CXCL10,IFI44,IFIT1B,Oasl2,STAT1

Table S5. Attempts to isolate replication competent virus from foot tissues of CHIKV infected C57BL/6 mice $\geq$30 days post infection. * positive when day 14 feet were used.

Isolation method	Amplification method	Detection assay	Outcome
Macerated foot tissue	C6/36	Vero CPE	Negative
Cells from collagenase digested macerated foot tissues	C6/36	Vero CPE	Negative*
Macerated and freeze thawed foot tissue	C6/36	Vero CPE	Negative
Supernatant from homogenised foot tissue	i.p. into IRF3/7$^{-/-}$ mice	Disease (Rudd et al., 2012)	Negative
Cells from macerated foot tissue	i.p. into IRF3/7$^{-/-}$ mice	Disease	Negative
Supernatant from homogenised foot tissue	i.p. into Rag1$^{-/-}$ mice	Viraemia	Negative
Cells from macerated foot tissue	i.p. into Rag1$^{-/-}$ mice	Viraemia	Negative
RNA isolated from foot tissues	Transfection of BHK cells	C6/36/Vero CPE	Negative

Figure S1A. Foot swelling and/or viraemia in C57BL/6 mice infected with CHIKV isolates recovered from Rag1$^{-/-}$ mice days 100 and 429 post infection. (a) Foot swelling in C57BL/6 mice inoculated with parental virus or virus derived from Rag1$^{-/-}$ mice 100 days post infection (Rag100). (n=8 feet per group). (b) Viraemia in C57BL/6 mice inoculated with parental virus or virus derived from Rag1$^{-/-}$ mice 100 days post infection (Rag100). (n=4 mice per group). (c) Viraemia day 1 and 3 in C57BL/6 mice inoculated with parental virus or virus derived from 3 different Rag1$^{-/-}$ mice 429 days post infection (Rag429). (n=4 mice per group).

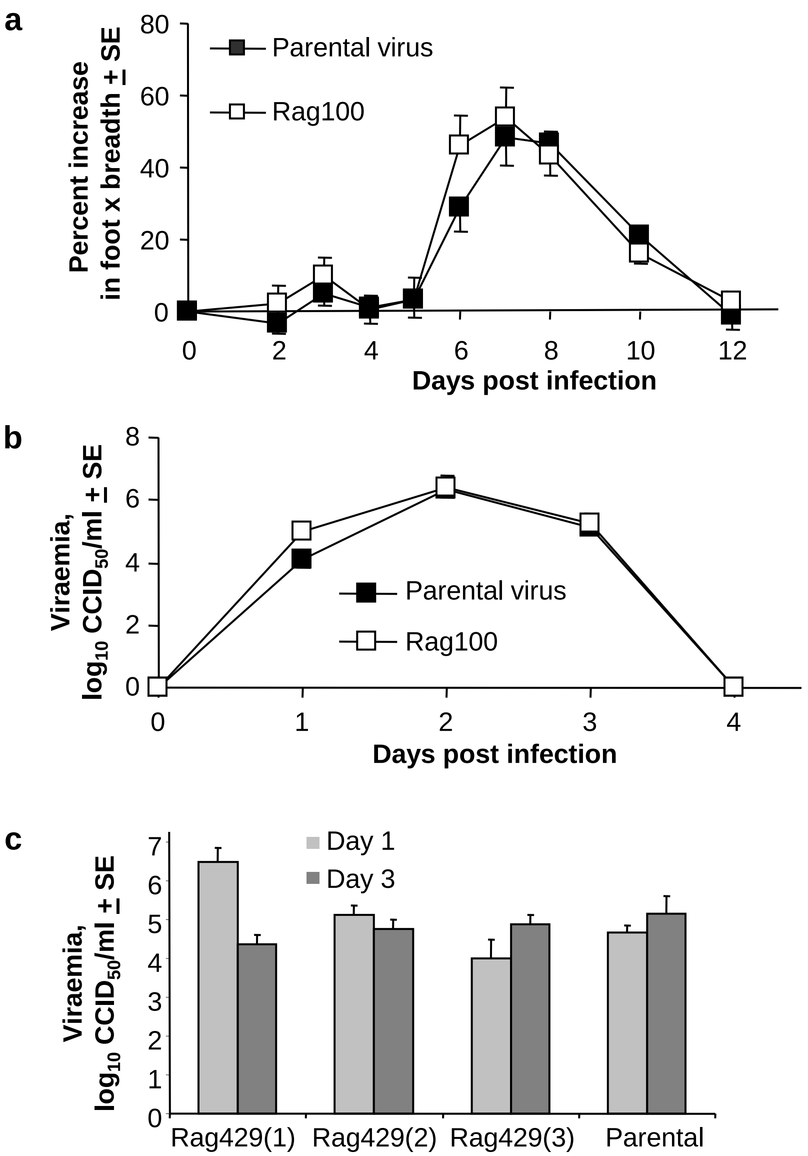

Figure S1B. Mutations identified in CHIKV recovered from Rag1-/- mice day 100 post infection. Parental Reunion Island isolate LR2006 OPY1 (passaged twice in C6/36 cells) used herein (Gardner et al., 2010) and virus isolated from Rag1$^{-/-}$ mice at day 100 post infection (Rag100) were grown in C6/36 cells, partially purified (0.22 uM filtration, DNase/RNase treated, and 100,000 g 1 h centrifugation), RNA isolated (QIAamp Viral RNA Mini Kit) and reverse transcribed, with the resultant viral cDNA amplified using the illustra GenomiPhi V2 DNA Amplification Kit (GE Healthcare). The sequence of the viral genome was then determined using semi-conductor sequencing (Ion Torrent PGM) at the Lotterywest State Biomedical Facility Genomics (University of Western Australia). Ion 314 chips were used to generate >200,000 reads of $\approx$100 bp. The sequence was assembled using Geneious Pro software and aligned with LR2006 OPY1 (Accession: DQ443544.2). Sequence changes are indicated below, changes highlighted in red. Percentages indicate the number of reads with the indicated nucleotide (threshold set at 2%) Three amino acid coding changes were identified.

Nucleotide position	Virus	Nucleotide	Coding AA
776	LR2006 OPY1	A	K
	Parental	G	E
	Rag100	G	E
1052	LR2006 OPY1	A and G	M/V
	Parental	G	V
	Rag100	G	V
4167	LR2006 OPY1	A and G	D/G
	Parental	A	D
	Rag100	A	D
4302	LR2006 OPY1	A	Y
	Parental	A	Y
	Rag100	A(34%) & G(65%)	C (nsP3^{1409})
5049	LR2006 OPY1	G and T	R/I
	Parental	T	I
	Rag100	T	I
5903	LR2006 OPY1	C	L
	Parental	C	L
	Rag100	C(34%) & T(66%)	L
6529	LR2006 OPY1	A	V
	Parental	A	V
	Rag100	A(72%) & G(28%)	V
8978	LR2006 OPY1	A	Q
	Parental	A	Q
	Rag100	G (96%)	R (E2^{471})
9203	LR2006 OPY1	A	K
	Parental	A	K
	Rag100	A(42%) & G(58%)	K and R (E2^{546})
11073	LR2006 OPY1	A	L
	Parental	A	L
	Rag100	A(26%) & G(74%)	L
3' UTR; 11395 & 11421 & 11455			
	LR2006 OPY1	C	
	Parental	C	
	Rag100	C (13,27,33%) & T (87,73,67%)	

Figure S1C. Integrated Genomics Viewer (IGV version 2.3.34 (41)) display of all the reads from the Ion Torrent output (map quality threshold 20, coverage allele frequency threshold 0.02). Coloured vertical lines on upper grey coverage graphs (log scale) match the nucleotide sites described in Fig. S1A. Mutations supported by 50-3000 independent reads, except UTR mutations supported by 6-15 independent reads. Lower black bars represent squished view of all Ion Torrent reads aligned to the CHIKV LR2006-OPY1 genome.

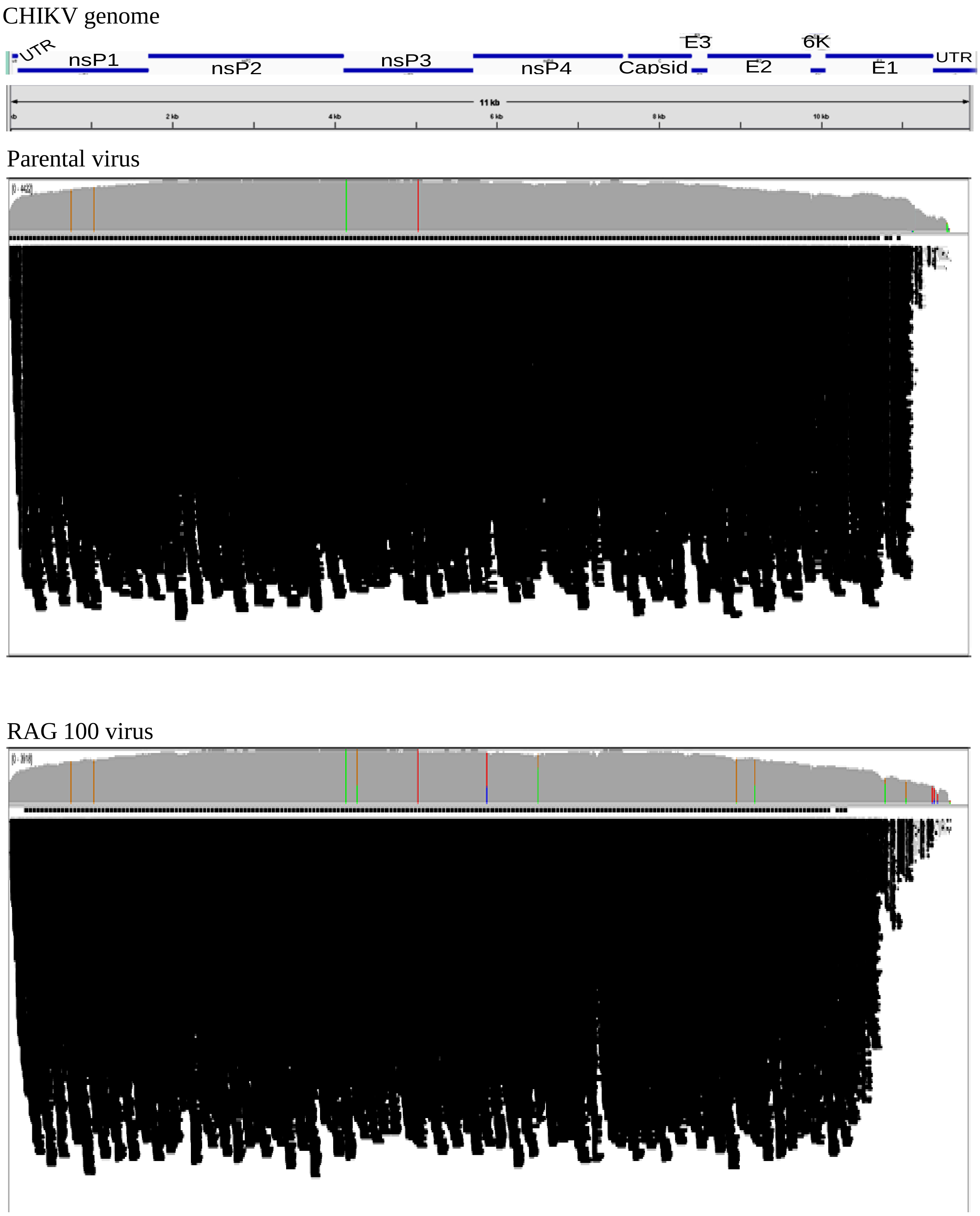

Figure S2A. H&E staining of feet after CHIKV infection of NOD mice showing inflammatory infiltrates in synovial membrane, muscle and dermis. Features are similar to those reported previously for C57BL/6 mice (Gardner et al., 2010). (a) Inflammatory infiltrate in the synovial membrane in NOD mice day 7 post infection. (b) Inflammatory infiltrate in muscle in NOD mice day 7 post infection. (c) Inflammatory infiltrate in dermis in NOD mice day 7 post infection. (* edema).

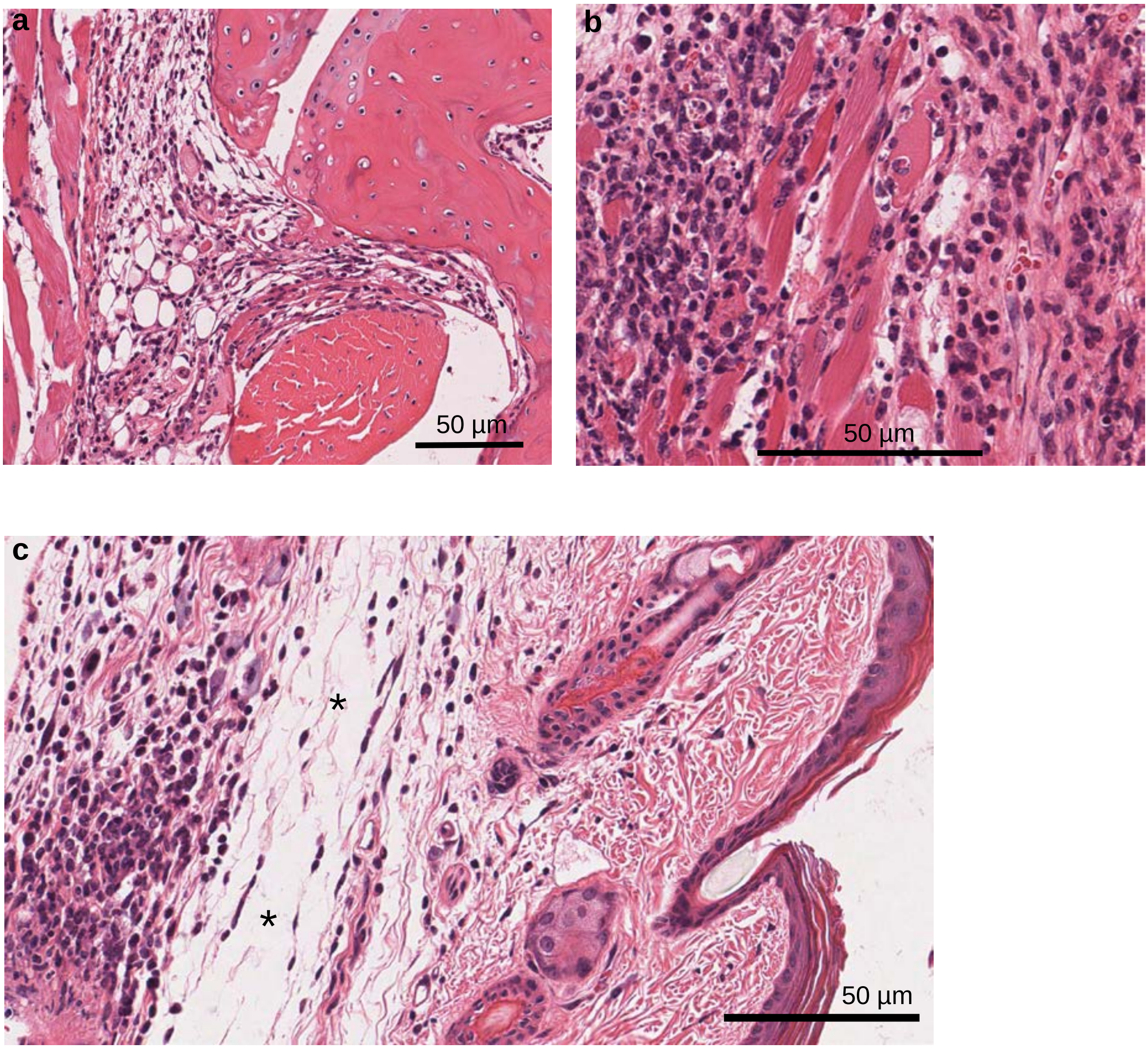

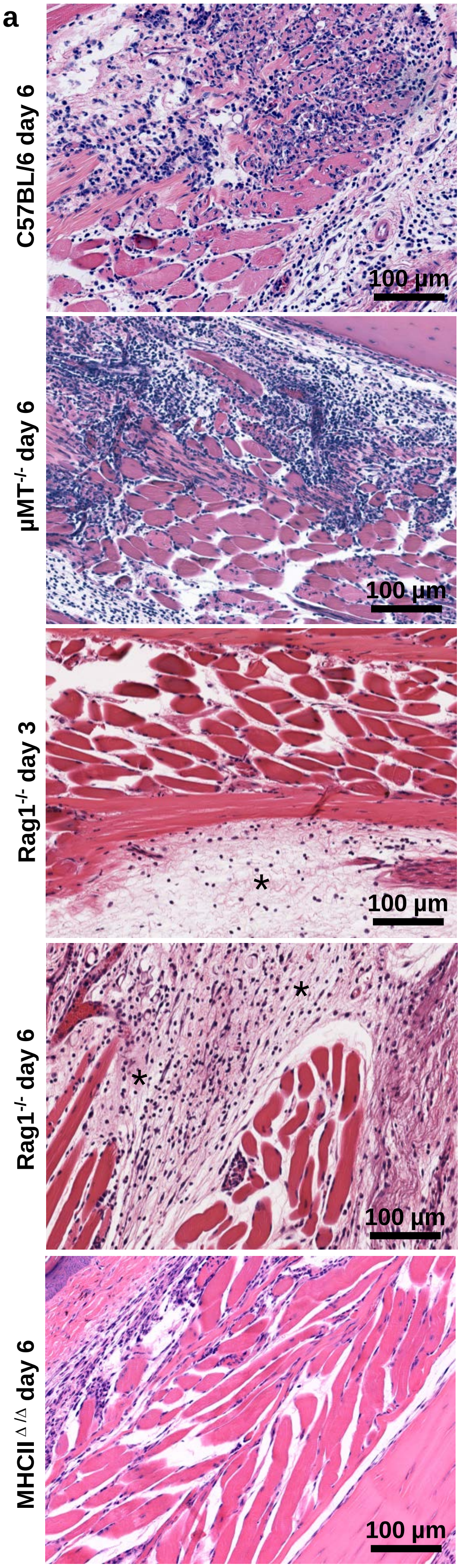

Figure S2B. Quantitation of cellular infiltrates in C57BL/6, Rag1$^{-/-}$, μMT, and MHCII $^{\Delta/\Delta}$ mice. (a) H&E staining of feet from the indicated mouse strains at the indicated times. (*) indicated edema.

(b) Image analysis of digitally scanned whole foot sections (as described Gardner et al 2010) using Aperio ImageScope Software (v10) and the Positive Pixel Count v9 algorithm (Strong blue, default settings). Strong blue measures nuclear staining and is thus a measure of the number of infiltrating cells. Mean of n = 4 feet per are shown. Statistics by Mann Whitney U and Kolmogorov Smirnov tests.

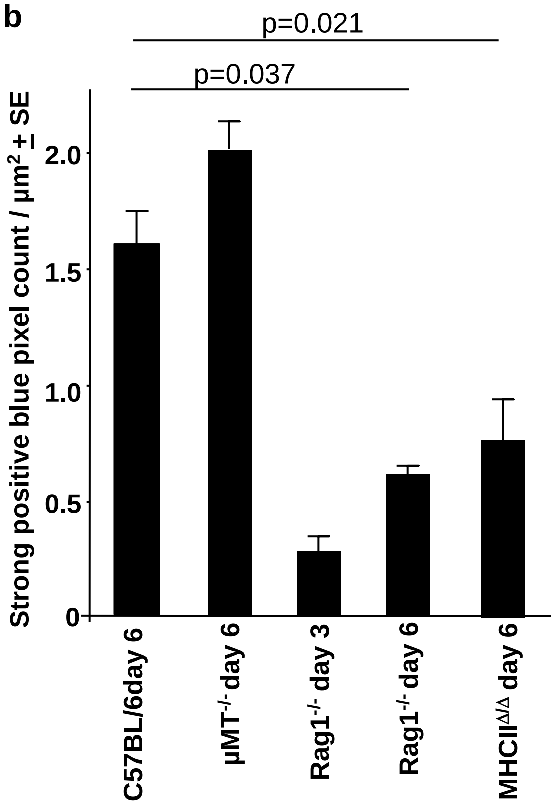

Figure S3. Early loss of viraemia control in B cell deficient mice and neutralising antibody responses in C57BL/6 mice. (a) Viraemia in B cell-deficient C57BL/6 mouse strains begins to be higher than C57BL/6 mice as early as day 3 post infection. This graph uses a subset of the data shown in Fig. 1, with data for the 4 indicated mouse strains plotted on one graph and the x axis restricted to day 0 to day 7. (b) Neutralisation titre against CHIKV in C57BL/6 mice determined as described (Wang et al. 2011. Vaccine 29;2803-2809); n=3-6 mice per time point.

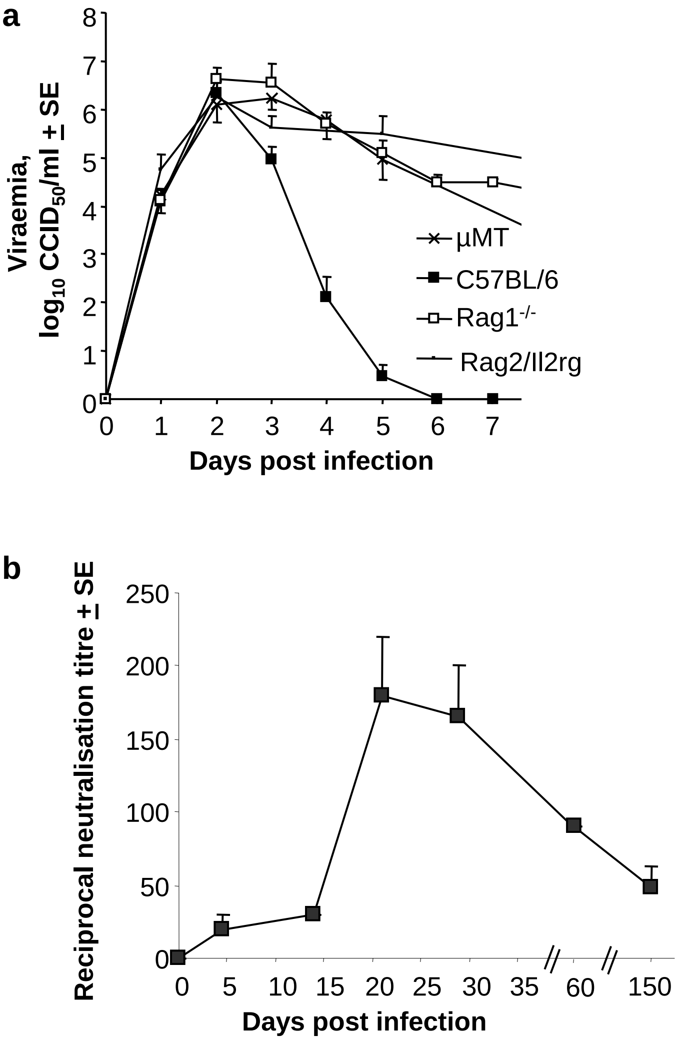

Figure S4. Viraemia and foot swelling in FcγR$^{-/-}$ mice**.** FcR-common gamma chain-deficient mice (FcγR$^{-/-}$) on a C57/BL6 background (Taconic, B6.129P2-Fcer1g^{tm1Rav} N12) were infected with CHIKV and viraemia (left) and foot swelling (right) measured. The common gamma chain (FcRγ) is required in mice for expression of all FcγR, except the inhibitory FcγRIIB.

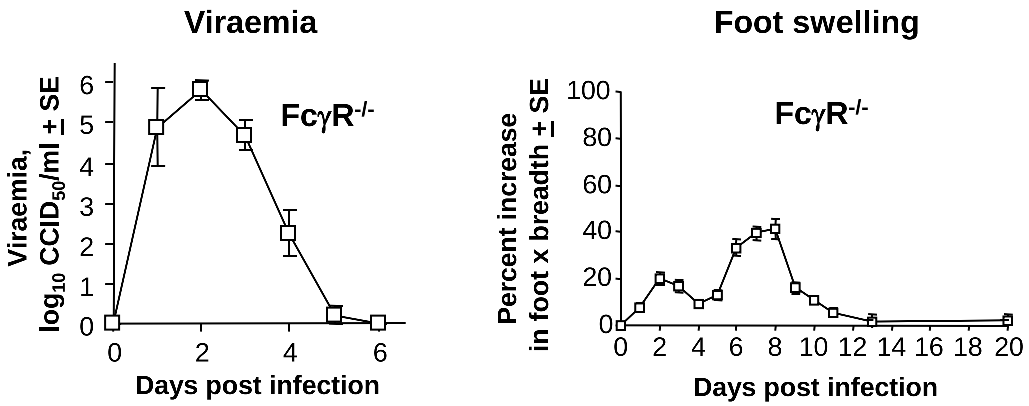

Figure S5. Histopathological modifications in spleen of chronically CHIKV infected Rag1$^{-/-}$ mice**.** H&E staining of spleen of uninfected Rag1$^{-/-}$ mice (left hand panels) and chronically infected Rag1$^{-/-}$ mice, day 430 post infection (right hand panels). The spleens of uninfected mice contained few polymorphonuclear leukocytes, whereas in the chronically infected mice granulocytosis (increased numbers of polymorphonuclear leukocytes) and granulopoiesis (as demonstrated by the presence of numerous doughnut-shaped cells) was evident.

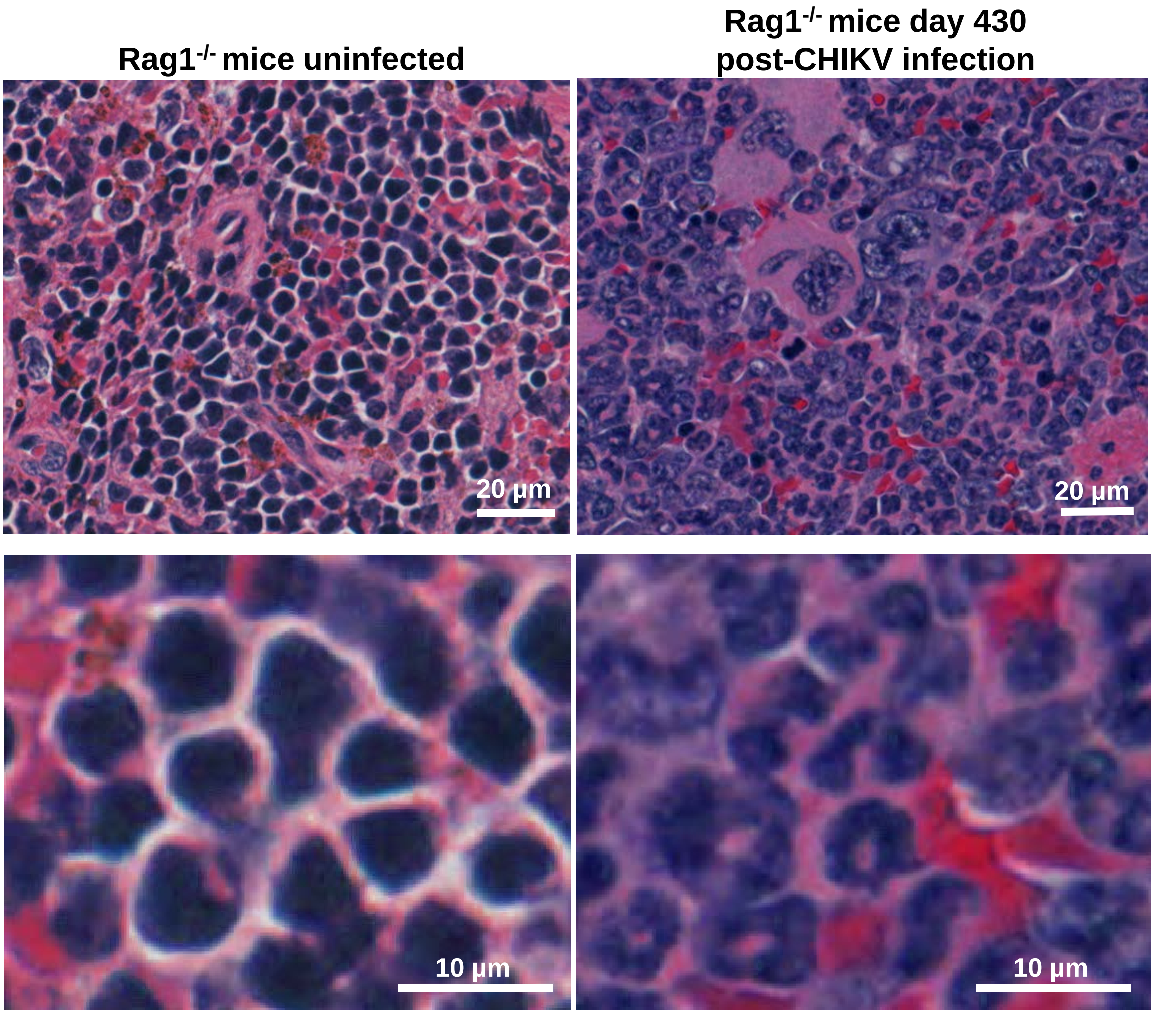

Figure S6. Survival of NRG mice post CHIKV infection.   Kaplan Meier survival plot of infected NRG mice. Mice were euthanised according animal ethics guidelines when symptoms were considered obvious but moderate. n=5 mice per group.

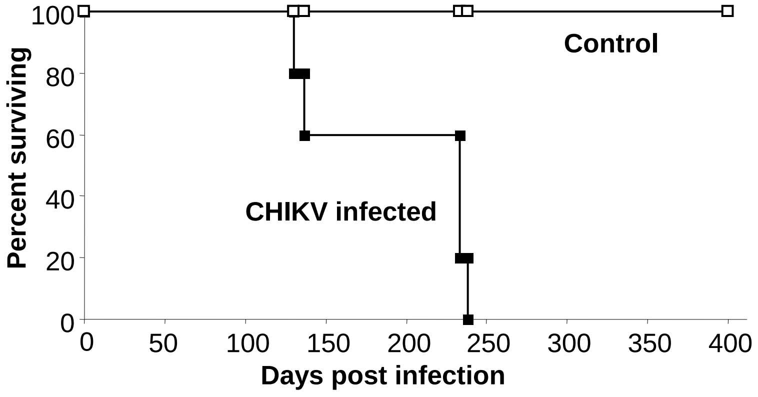

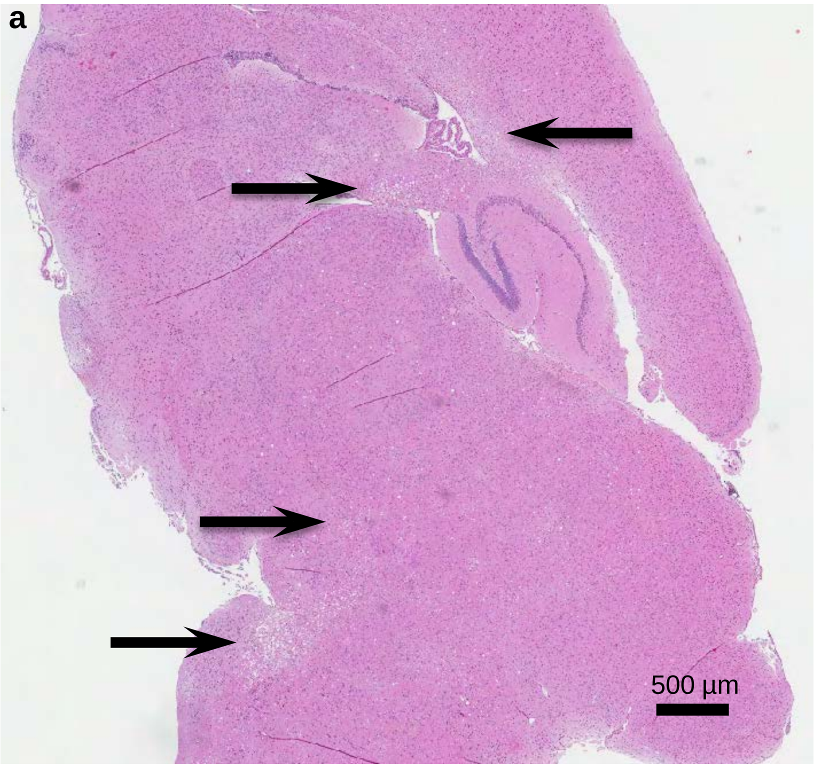

Figure S7A. Brain lesions in NRG mice with neurological symptoms requiring euthanasia. (a) Low magnification H&E staining of the brain of NRG mouse with CHIKV-induced neurological symptoms requiring the animals to be euthanasized. Arrows indicate pale foci near ventricles (corpus callosum, striatum), in the thalamus, and in Ammon's horn. (No additional lesions were seen with Luxol Fast Blue staining: there was no evidence of demyelination). (b) High magnification of a pale foci, showing the foci correspond to areas of vacuolisation and edema. The cell density depicted here is illustrative of diffuse reactive gliosis. (c) In or near the vacuolated foci gemistocytic astrocytes, with abundant hyalin eosinophilic cytoplasm and large clear nucleus, were evident (black arrowhead). Some activated microglial cells were also present (arrows). Dark staining neurons within the damaged parenchyma (suggestive of cell death) were also seen (open arrowhead).

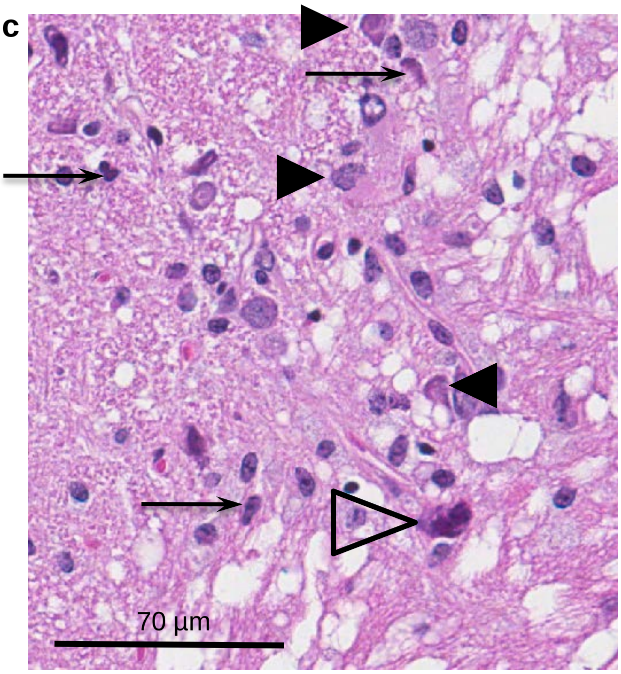

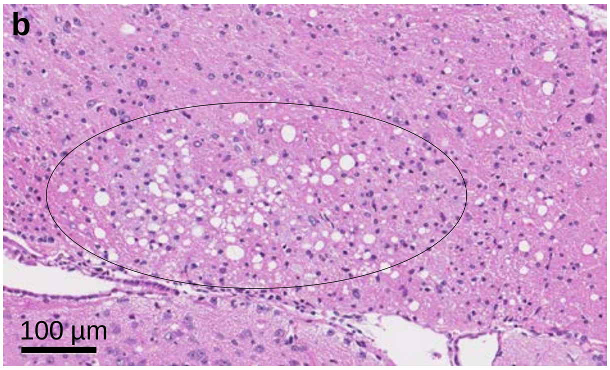

Figure S7B. Immunohistochemical staining of CHIKV capsid protein in brain tissue of CHIKV infected NRG mice with neurological symptoms requiring euthanasia day 228 post infection. Paraffin sections were stained with a new anti-CHIKV monoclonal antibody (5.5G9) that recognizes the CHIKV capsid protein (Goh et al, submitted). Red staining (chromogen-substrate, 3-amino-9-ethylcarbazole) indicates the presence of capsid antigen. Staining protocol as described in Goh et al. 2013 (Clin Immunol. 149(3):487-97). (a) CHIKV positive cells in Ammon's horn, with morphology consistent with neurons and oligodendrocytes. (b) CHIKV positive cells in the Bulbus olfactorius, with morphology consistent with neurons. (c) CHIKV positive cells in the white matter, morphology consistent with oligo-dendrocytes (d) Isotype control, NRG mouse brain day 228 post infection. (e) Isotype control, NRG mouse brain day 228 post infection. (f) Aperio ImageScope software (v10) and the Positive Pixel Count v9 algorithm was used as described (Poo et al, 2014) to quantitate red capsid staining. Anti-capsid antibody staining showed ≈100 fold more red pixels than staining of the same tissue with an isotype control or anti-capsid staining of brain from C57BL/6 mice, which do not show neurological symptoms.

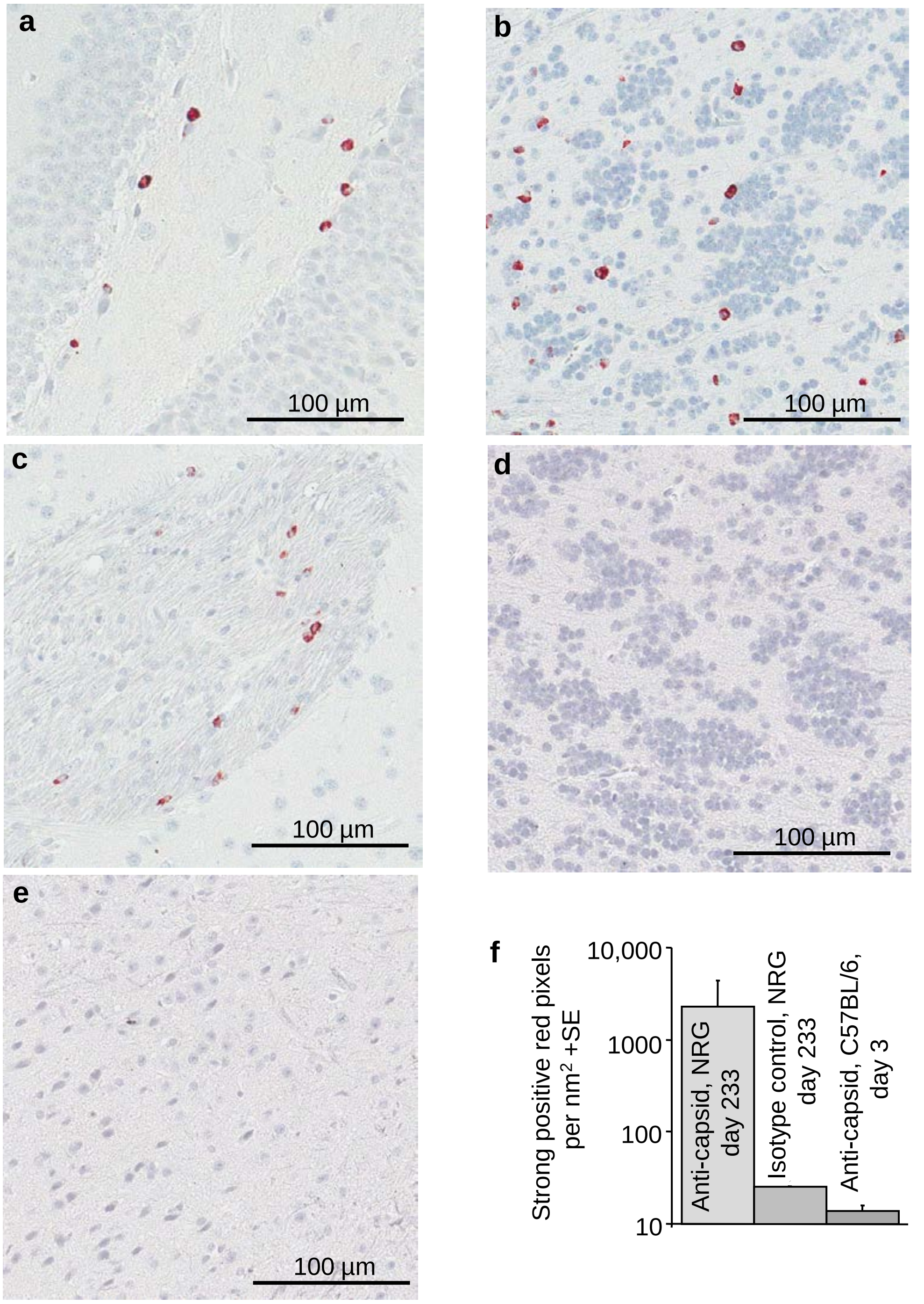

Figure S8A. No persistent CHIKV RNA in spleen. qRT-PCR analysis of whole spleens taken at the indicated times post infection and analysed as for Fig. 5A and at the same time as samples from feet (to provide positive controls). n= 6, 2, 4 and 4 for the indicated days, respectively.

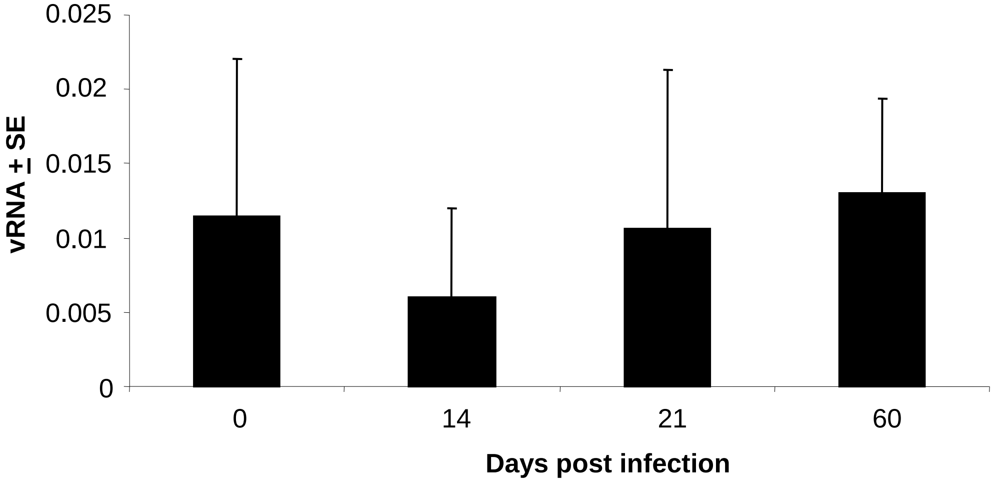

Figure S8B. Curve fitting of decline in persistent post-viraemic CHIKV RNA in wild-type mouse feet over time. CHIKV RNA data from Figure 5A and B, showing viraemic period (yellow) and curve fit for (i) RNA levels (measured by standard qRT PCR) and (ii) negative strand RNA (-RNA) levels, from day 14 onwards. Day 100 for -RNA was excluded as an outlier. (Distinct methods were used to measure RNA and -RNA, thus the different y values for RNA and -RNA cannot be used to compare RNA and -RNA levels. Similarly, where y=ae-x, differences in "a" reflect differences in the methods used).

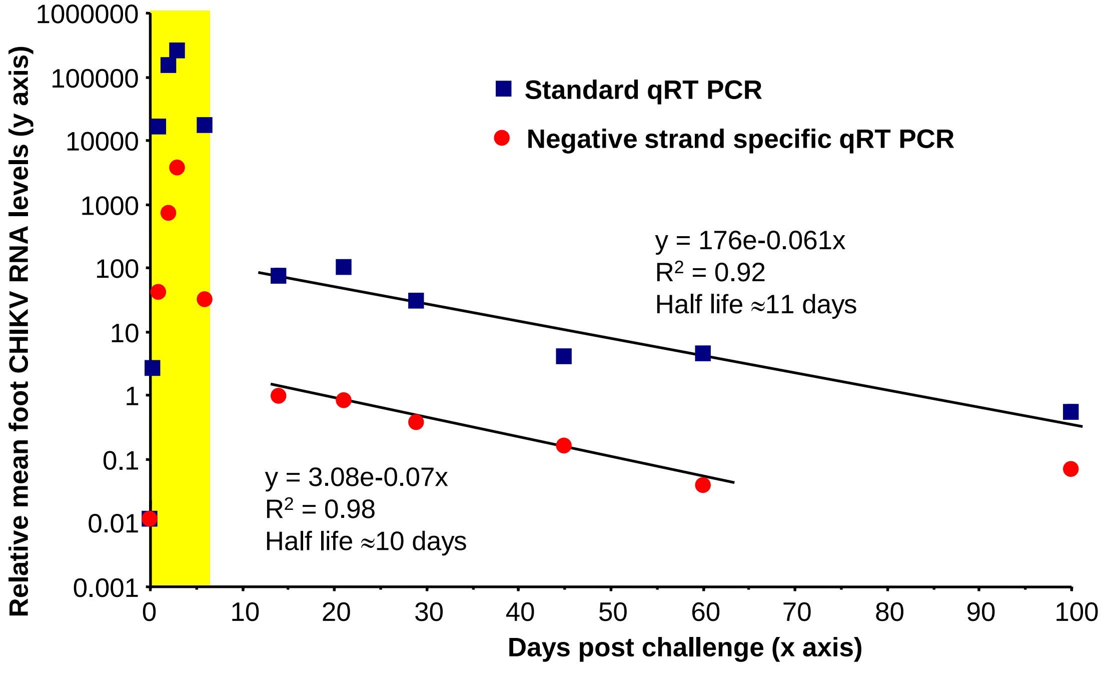

Figure S9. Persistence of viral antigen in feet of CHIKV infected mice. Immunohistochemical staining of paraffin sections of feet with new anti-CHIKV capsid monoclonal antibody (5.5G9) as described (Goh *et al*. submitted). Red staining indicates presence of antigen; 9 images are shown for day 30 (same magnification for all images) (left), isotype control and an uninfected control are shown (right).

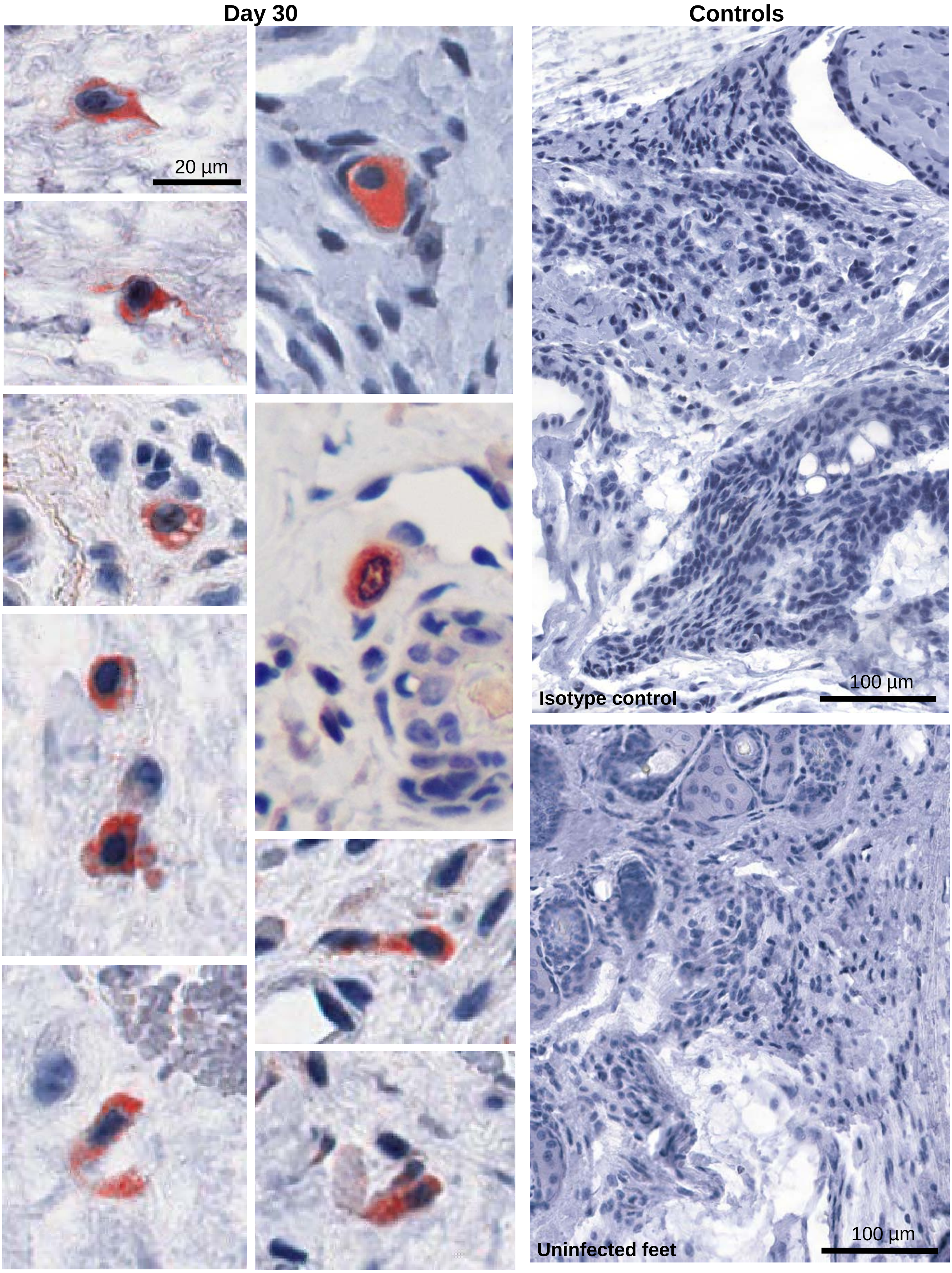

Figure S10A. Principal component analysis of 4,805 filtered genes identified by microarray analysis of feet of wild-type mice day 0 and 30 post infection. Microarray analysis was conducted as described (Nakaya et al, 2012) comparing feet from day 0 and day 30 post-infection with CHIKV. Probe sets that did not represent known genes were removed and only expressed genes with a mean $\log_2$ expression $\geq$ 6 and variance > 0.1 across all 4 samples were included. The former removed genes with overall low expression. The later removed genes whose expression changed little regardless of infection and were thus deemed uninformative. Expression was normalized by z-score. Red squares – two day 30 samples (from 3 pooled feet), black squares - two day 0 samples (from 3 pooled feet). 4,805 filtered genes were used for the PCA; 1,000 clusters were used for PCA calculation.

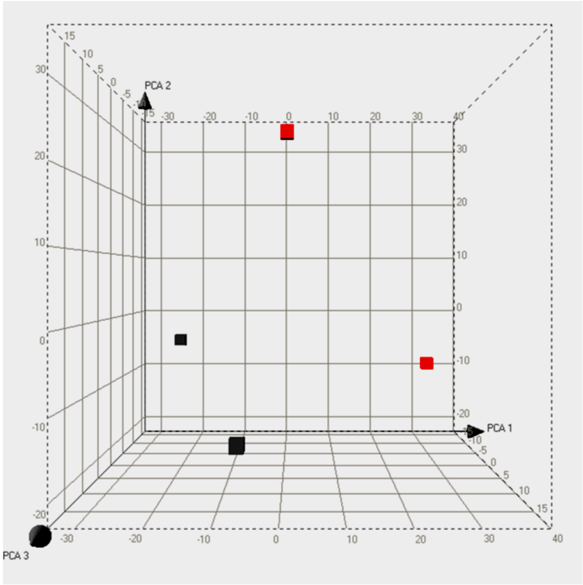

Figure S10B. Heat map of 192 significantly up-regulated genes identified by microarray analysis of feet of wild-type mice day 30 post infection. Of the 4,805 fitered genes identified above, 192 genes were significantly up-regulated on day 30 compared with day 0 (t test $p < 0.05$).   A heat map of the 192 genes identified is shown. No FDR was performed.

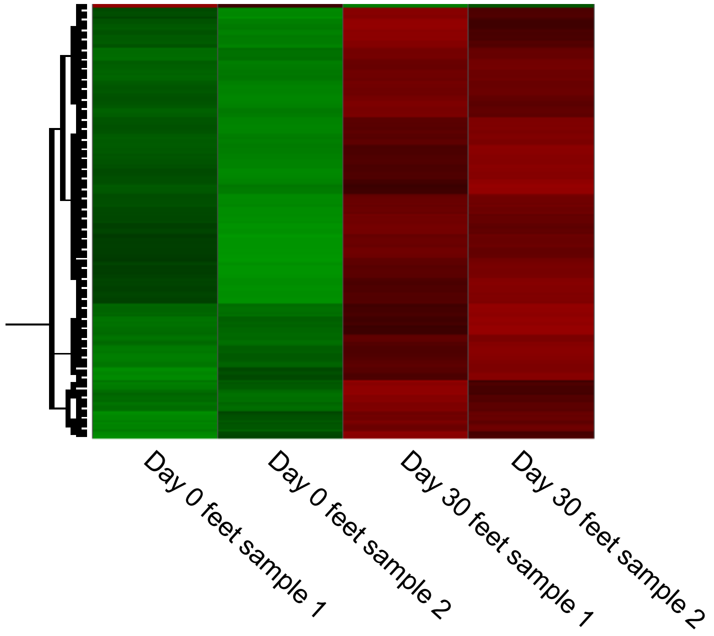

Figure S10C. qRT PCR of Granzyme B and FcγR4 at days 0, 7 and 30 post infection with CHIKV. n=3-6 feet/mice per time point. Statistics by Kolmogorov-Smirnov test. Primer sequences: GranB, F 5'- CAC TCT TGA CGC TGG GAC C -3‘, R 5'- GAG CAG CAG TCA GCA CAA AG -3; FcγR4 F 5'- TTC AGC AGC ATG TGG CAG -3‘, R 5'- AAC CAC TTG ATA GAA TTG TCC TCG -3‘.

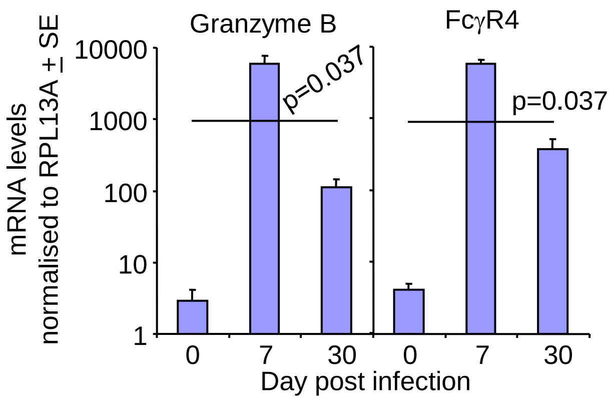

Figure S11. Ingenuity canonical pathway analysis of the 192 genes up-regulated in feet of mice at day 30 post infection. The pathways have been grouped into themes as described previously for pathways identified on day 7 post infection (Nakaya et al. 2012). -log (p value) > 1.3 represents $p < 0.05$.

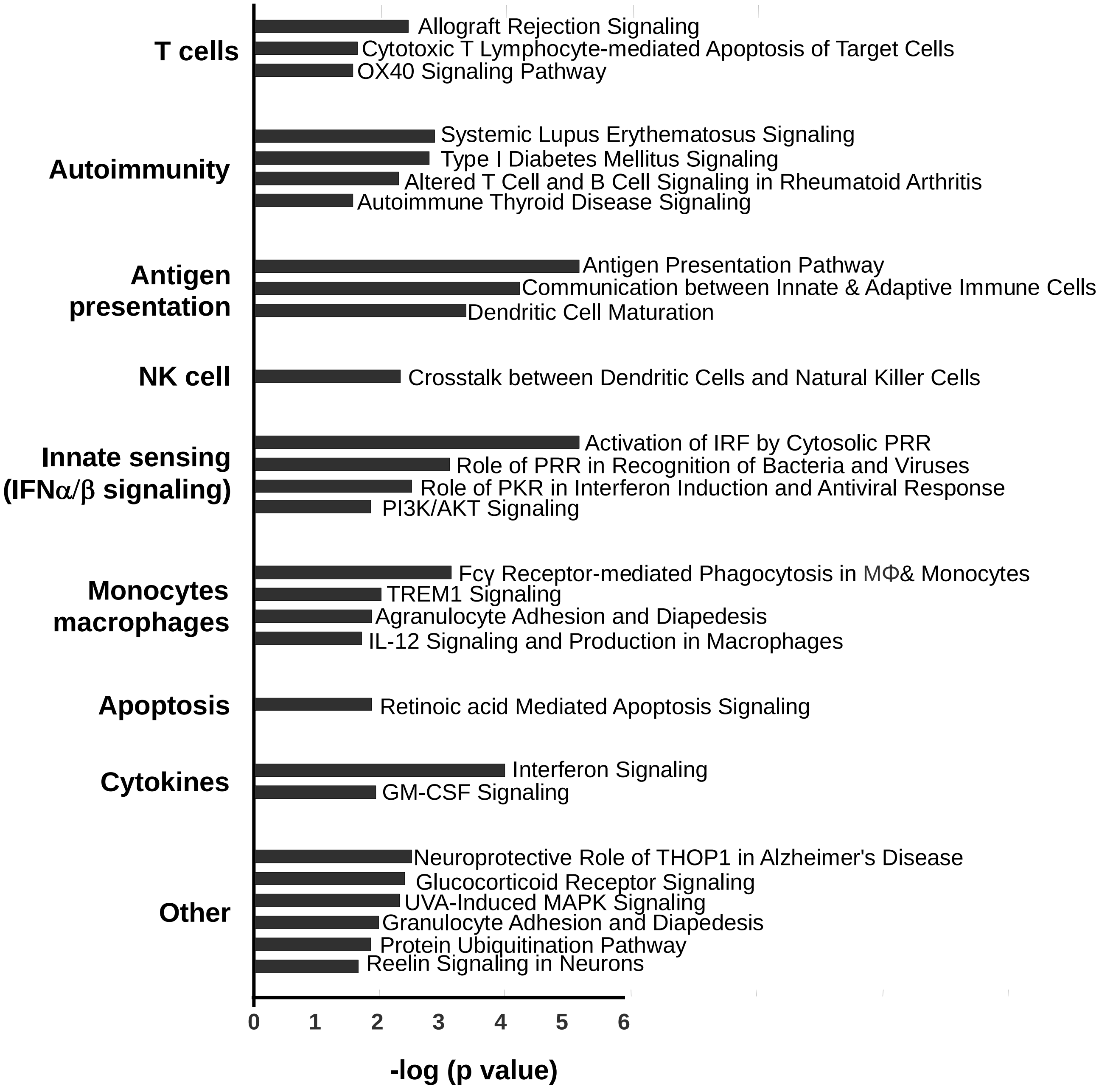

Supplement: Text S1 — This file contains supporting information tables and figures. Table S1. The effect of age of mice within a 6–12 week old age range on peak foot swelling. Table S2. List of all the mice strains used in the current study. Table S3. Table of 192 genes identified by microarray as up-regulated in feet on day 30 post infection. Table S4. Table showing the full data set for upstream regulators shown in Table 1. Table S5. Attempts to isolate replication competent virus from foot tissues of CHIKV infected C57BL/6 mice≥30 days post infection. Figure S1 A. Foot swelling and/or viraemia in C57BL/6 mice infected with CHIKV isolates recovered from Rag1−/− mice days 100 and 429 post infection. Figure S1 B. Mutations identified in CHIKV recovered from Rag1−/− mice day 100 post infection. Figure S1 C. IGV display of deep sequencing output for parental and Rag100 viruses. Figure S2 A. H&E staining of feet after CHIKV infection of NOD mice showing inflammatory infiltrates in synovial membrane, muscle and dermis. Figure S2 B. Quantitation of cellular infiltrates in C57BL/6, Rag1−/−, µMT, and MHCII Δ/Δ mice. Figure S3. Early loss of viraemia control in B cell deficient mice, and neutralising antibody responses in C57BL/6 mice. Figure S4. Viraemia and foot swelling in FcγR−/− mice. Figure S5. Histopathological modifications in spleens of chronically CHIKV infected Rag1−/− mice. Figure S6. Survival of NRG mice post CHIKV infection. Figure S7 A. Brain lesions in NRG mice with neurological symptoms requiring euthanasia. Figure S7 B. Immunohistochemical staining of CHIKV capsid protein in brain tissue of NRG mice with neurological symptoms requiring euthanasia. Figure S8 A. No persistent CHIKV RNA in spleen. Figure S8 B. Curve fitting of decline in persistent post-viraemic CHIKV RNA in wild-type mouse feet over time. FigureS9. Persistence of viral antigen in feet of CHIKV infected mice. Figure S10 A. Principal component analysis of 4,805 filtered genes identified by microarray analysis of fe [file pntd.0003354.s001.pdf]
